# RNA sequencing demonstrates large-scale temporal dysregulation of gene expression in stimulated macrophages derived from MHC-defined chicken haplotypes

**DOI:** 10.1371/journal.pone.0179391

**Published:** 2017-08-28

**Authors:** Kristopher J. L. Irizarry, Eileen Downs, Randall Bryden, Jory Clark, Lisa Griggs, Renee Kopulos, Cynthia M. Boettger, Thomas J. Carr, Calvin L. Keeler, Ellen Collisson, Yvonne Drechsler

**Affiliations:** 1 College of Veterinary Medicine, Western University of Health Sciences, Pomona, California, United States of America; 2 The Applied Genomics Center, Graduate College of Biomedical Sciences, Western University of Health Sciences, Pomona, California, United States of America; 3 College of Veterinary Medicine, Michigan State University, East Lansing, Michigan, United States of America; 4 Department of Biological Sciences, University of Delaware, Newark, Delaware, United States of America; 5 Department of Biological Sciences, Northern Illinois University, DeKalb, Illinois, United States of America; Kunming University of Science and Technology, CHINA

## Abstract

Discovering genetic biomarkers associated with disease resistance and enhanced immunity is critical to developing advanced strategies for controlling viral and bacterial infections in different species. Macrophages, important cells of innate immunity, are directly involved in cellular interactions with pathogens, the release of cytokines activating other immune cells and antigen presentation to cells of the adaptive immune response. IFNγ is a potent activator of macrophages and increased production has been associated with disease resistance in several species. This study characterizes the molecular basis for dramatically different nitric oxide production and immune function between the B2 and the B19 haplotype chicken macrophages.A large-scale RNA sequencing approach was employed to sequence the RNA of purified macrophages from each haplotype group (B2 vs. B19) during differentiation and after stimulation. Our results demonstrate that a large number of genes exhibit divergent expression between B2 and B19 haplotype cells both prior and after stimulation. These differences in gene expression appear to be regulated by complex epigenetic mechanisms that need further investigation.

## Introduction

Discovering genetic biomarkers associated with disease resistance and enhanced immunity is critical to developing advanced strategies for controlling viral and bacterial infections in various species.

Disease resistance and susceptibility depends on a variety of factors including genetics. In numerous species, disease resistance has been associated with major histocompatibility complex (MHC) haplotype, as well as polymorphisms in several immune genes such as TGFβ and TNFα[1,2]. Cytokine production, specifically secretion of pro-inflammatory molecules, has also been associated with increased resistance against disease [3,4].

Studies have demonstrated association of MHC-B haplotype in chickens and resistance to a variety of viral pathogens, including AIV, Marek’s disease virus (MDV), avian leukosis virus, Newcastle disease virus and Rous sarcoma virus [5–10] as well as other pathogens [11,12]. B2 haplotype chickens are more resistant to avian coronavirus infection than B19 haplotypes and these differences in disease resistance were observed early after infection in our previous studies [10]. This suggests that innate immunity plays a major role with the macrophage being a key player in this enhanced immune response as evidenced by the B2 haplotype birds’ greater capability to produce nitric oxide (NO) in response to IFNγ and Poly I:C [13]. In addition, B2 macrophages activated T cells more efficiently than macrophages derived from B19 haplotypes [14].

Macrophages are directly involved in cellular interactions with pathogens and demonstrate distinct immune responses from more disease resistant animals in response to infection [15–20]. In addition, macrophages release cytokines activating other immune cells and antigen presentation to cells of the adaptive immune response [21–23]. It has become increasingly clear that dysregulation of macrophage function is involved in inflammatory disease processes such as rheumatoid arthritis, inflammatory bowel disease and cancer [24–26]. Involved in these interactions are crucial molecules such as Toll-like receptors (TLRs) that recognize invading microorganisms, resulting in communication with the adaptive immune system such as increased expression of MHC surface molecules, T cell receptors and secreted cytokines [21,23]. Genetic differences in any of those molecules can potentially account for differences in immune competence and thus provide potential immunogenetic markers for disease resistance to various pathogens.

IFNγ is a potent activator of macrophages and increased production has been associated with disease resistance in multiple species [27–31]. These findings indicate that chickens with enhanced IFNγ production are more resistant to certain infections. IFNγ enhances macrophage activation, expression of MHC and nitric oxide release which aides in killing of pathogens and also increases activity of cytotoxic T cells and secretion of Th1 cytokines [31,13], underscoring how crucial this process is for innate immune competence.

Macrophage TLRs appear to be primed by IFNγ, reprogramming cellular responses to other cytokines, such as type I interferons and IL-10 and activating the Jak-STAT pathway (Janus kinase and signal transduction and activator of transcription) [24, 32, 33]. IFNγ, which increases TLR receptor availability for interaction with its ligands, has been shown to induce TLR2, 4, 6 and 9 [34–37].

The response of macrophages to an immune stimulus is not just dependent on cell surface receptor and cytokine expression. Other factors include the differentiation of monocytes into functional macrophages, a tightly regulated process that influences immune competence [38]. Recent studies demonstrated a critical role for molecules such as A2B adenosine receptor for differentiation and proliferation of monocytes and macrophage function in immunity and inflammation [39, 40]. A2B expression is induced by IFNγ and leads to increase of anti-inflammatory signaling counteracting the inflammatory response activated within the IFNγ pathway.

Taken together, these studies emphasize the genetic basis of the activation of macrophages by IFNγ playing an important role in the innate immune response signaling and providing resistance to disease. In addition to inflammatory signaling, a number of transcription factor pathways and epigenetic mechanisms all contribute to immune function. Dysregulation of any of these events will lead to an impaired innate immune response and consequently, increased susceptibility to disease.

Using an *ex vivo* model, we investigated the gene expression in macrophages from haplotypes B2 and B19 during differentiation and after stimulation with IFNγ. Our experimental design leveraged an initial 6 day window for monocytes to differentiate into macrophages, which was followed by IFNγ stimulation between 1 and 24 h to further characterize subsequent RNA gene expression and the molecular basis for dramatically different nitric oxide production and immune function between the B2 and the B19 haplotype chicken macrophages

## Material and methods

### Experimental animals

Animal protocols were performed under the approval of the Institutional Animal Care and Use Committee at Western University of Health Sciences, Pomona, California (WesternU). Fertilized eggs, descended from Modified Wisconsin Line 3, were obtained from Dr. W. Elwood Briles, Northern Illinois University, and incubated and hatched under standard conditions at (38°C/50-65% humidity) [10,13] at WesternU. In addition to daily health monitoring, fresh food and water were provided *ad libitum*. Experimental animals were euthanized by insufflation of isoflurane gas (Butler, Dublin, OH).

### Peripheral blood collection

Whole blood samples were collected via jugular venipuncture in EDTA tubes from age matched chicks at 14–18 weeks old. At no time did the amount of blood harvested from each animal exceed 1% of body weight.

### Peripheral blood mononuclear cell (PBMC) isolation

Peripheral blood mononuclear cells (PBMCs) from individual birds were isolated using the differential centrifugation as previously described [41, 42, 13] with slight modifications. Briefly, blood was mixed with an equal volume of phosphate buffered saline (PBS) and slowly layered 2:1 on a Ficoll-Hypaque gradient (density 1.083) (Sigma-Aldrich, St. Louis, MO). Samples were centrifuged for 35 min (400 x g; 23°C; brake off) for retrieval of mononuclear cells. Isolated cells were washed 3x in 10 ml PBS at low speed to remove thrombocytes (180 x g; 10 min, 23°C), counted and viability confirmed based on the exclusion of 0.1% trypan blue dye (≥ 90%). PBMCs were re-suspended in PBS to a final concentration of 5 x 10^7^ cells/ml.

### Macrophage cell culture

One milliliter of PBMC suspension (5 x 10^7^ cells/ml) was incubated (37°C/5% CO_2_) for 3 h in each well of a 12-well plate containing RPMI w/o Phenol Red supplemented with, 10% heat inactivated fetal bovine serum (FBS); non-essential amino acids, (0.1mM/ml) (Invitrogen, Carlsbad, CA), L-glutamine (2 mM) (Sigma-Aldrich, St. Louis, MO), 2-mercaptoethanol (55 μM/ml) (Sigma-Aldrich, St. Louis, MO), penicillin (50 U/ml) (Invitrogen, Carlsbad, CA), and streptomycin (50 μg/ml) (Invitrogen, Carlsbad, CA). Following removal of non-adherent cells with warm PBS, medium was replenished and cells were incubated for differentiation with the exception of the -6 day sample which was lysed with 400 μl Trizol (Thermo Scientific, Waltham, MA) and stored at -80°C. Prior to the replacement of medium, adherent cell cultures were washed in warm PBS. Monocytes were cultured for 6 days to allow maturation and differentiation of cells; with medium changes occurring every 3–4 days thus ensuring that optimal nutrient requirements were met. Additionally, -3 day (t-3) samples were lysed with Trizol and stored at -80°C. The morphology of adherent cells was observed daily under bright field microscopy (20x objective).

Purity of monocyte cultures using this culture method was confirmed by IFA and FACS using monoclonal antibody KUL01 as previously described as part of a different aspect of this study [13].

### IFNγ stimulation

A 50 ρg/ml ch-IFNγ solution (Invitrogen, Carlsbad, CA) was prepared in RPMI w/o Phenol Red culture medium (Invitrogen, Carlsbad, CA). After washing the cells twice with warm PBS, macrophage cultures were stimulated with 1 ml of RPMI-ch-IFNγ mixture [13].

### Nitric oxide assays

Nitric oxide production was measured [10, 43, 44] to confirm macrophage stimulation in assays by interferon (data not shown). Stimulation was evaluated as yes/no based on previously published results from B2 and B19 IFNγ stimulated macrophages (10)

### Sample collection and RNA sequencing

A total of 145 gigabytes of RNA sequence data was obtained from B19 and B2 haplotypes of chickens. Two birds from each haplotype were selected for inclusion in the sequencing. Each bird provided blood for extraction and isolation of peripheral blood mononuclear cells. Purified monocytes were cultured for differentiation and cell samples were collected from nine time points for each bird. Samples were collected for sequencing on the day they were cultured (Day t-6), as well as on Day -3 (t-3), Day 0 (called 0 hours), and then six additional times over a 24 hour period corresponding to 1 hour, 2 hours, 4 hours, 8 hours, 16 hours and 24 hours after interferon stimulation. Cells were lysed in wells with RLT buffer containing beta-mercaptoethanol (Qiagen, Valencia, CA) and stored at -80°C. RNA was processed with the Qiashredder and RNAeasy kit from Qiagen (Valencia, CA) according to manufacturer’s instructions and sent on dry ice to Dr. Calvin Keeler at the University of Delaware for generation of libraries and sequencing with an Illumina HiSeq 2000.

An RNA sequence library was constructed from purified RNA. The library was fragmented in order to generate appropriately sized RNA fragments suitable for templates in random primed first-strand cDNA synthesis. Second strand synthesis was completed in accordance with specifications for sequencing with Illumina’s HiSeq2000 platform.

The samples corresponding to each time point from each bird were sequenced and the data was stored in a unique file for each sequenced sample and time point. Forty FASQ files were generated from the data totaling 145 gigabytes. The average file size was 3.65 gigabytes and the standard deviation was 2.25 gigabytes. The sequencing data provided 933,107,885 reads across the biological samples and time points (Table 1). Across all time points for the two B2 samples, one produced 298,903,517 reads and the other produced 165,589,594 reads. Similarly, across all time points, the B19 samples produced 285,392,384 reads and 183,222,390. For each time point (across all four birds) sequencing reads ranged from a low of approximately 78 million reads to a high of just over 171 million reads, with most time points producing over 88.4 million reads each and a few producing over 100 million reads each.

**Table 1:** Sequencing reads across biological samples and time points.

		**t-6 days**	t-3 days	t0 h	t1 h	t2 h	t4 h	t8 h	t16 h	t24 h	TOTAL t-6d_to_t24h
**BIRD-A** **(B2)**	reads	25470757	13671735	8242127	23744417	47908759	78741373	71211600	23314834	6597915	298903517
	aligned reads	11471896	8872332	5508010	16979440	35666816	56923920	38573825	10060460	3421906	187478605
	multiple alignments	148334	43889	21791	70270	131735	229889	141032	49269	16332	852541
**BIRD-B** **(B2)**	reads	1459795	28536965	24829151	29604495	1806807	5394361	25715126	17422945	30819949	165589594
	aligned reads	87027	21502194	17672812	22534847	1268878	3911819	18816906	13460540	23620025	122875048
	multiple alignments	425	92783	63653	78722	6541	18257	86313	39605	92277	478576
**BIRD-C** **(B19)**	reads	44587849	32008868	21166414	25722656	23055824	25946021	59435090	29226936	24242726	285392384
	aligned reads	17419206	12412811	14534824	16720019	15511707	17668026	41819414	16704419	16847457	169637883
	multiple alignments	177899	114518	78430	99577	73310	65676	177083	75290	63288	925071
**BIRD-E** **(B19)**	reads	28452005	6000451	23444741	20988785	22333873	21392153	14715694	18485412	27409276	183222390
	aligned reads	10587715	775587	14000961	14095140	15379919	15685618	10520441	12590915	20507446	114143742
	multiple alignments	212095	1803	80898	73748	82985	28868	39248	62596	61264	643505
**ALL 4 BIRDS**	reads	99970406	80218019	77682433	100060353	95105263	1.31E+08	171077510	88450127	89069866	933107885
	aligned reads	39565844	43562924	51716607	70329446	67827320	94189383	109730586	52816334	64396834	594135278
	multiple alignments	538753	252993	244772	322317	294571	342690	443676	226760	233161	2899693

### Mapping reads to reference genome and identification of splice junctions / exons

The chicken reference genome WASHUC2, corresponding to Ensembl release 70, was downloaded from Ensembl.org (http://www.ensembl.org/info/data/ftp/index.html). Annotation files included the small RNA annotation files obtained from miBase release 19 (http://www.mirbase.org/). Sequenced reads were filtered to remove low quality sequences from the data. Filtered sequences were aligned to the reference genome using Bowtie and Tophat, available along with the software package Cufflinks, from John Hopkins University Center for Computational Biology (https://ccb.jhu.edu/software.shtml). The aligned reads generated by Bowtie produced gapped alignments on the reference genome which Tophat used to identify splice junctions flanking exons. The resulting aligned reads were analyzed by Cufflinks to construct transcripts corresponding to mRNA sequences. Next, Cufflinks was employed to estimate transcript specific expression levels across the transcripts and genes within the reference genome based on the number of sequence reads for each mRNA. The sequence read data was normalized using the fragments per kilobase of transcript per million mapped reads (FPKM) method to more accurately determine expression levels. The resulting transcriptome data was loaded into the MySQL relational database to more effectively manage, explore, mine and annotate the data.

### Hierarchical clustering of genes and production of heat map visualization

Gene expression data was hierarchically clustered using 1-Pearson correlation on the rows and keeping the column order conserved. The resulting clustered data set was visualized as a heat map with black representing lack of gene expression, and darker shades of blue indicating lower expression values. Dark purple represents higher expression values than any shade of blue while bright pink represents the highest expression values. For visualization purposes, the heat maps were generated with maximum heat map color assigned to expression set lower than the absolute maximum expression value contained in the entire data set, subsequently all values of expression greater than or equal to the assigned expression threshold (for example, 1000) shared the same color on the heat map (regardless of whether the actual expression level was 1000, 2000, 20,000 or 90,000). This setting provided the optimal visualization of both high and low expressed genes in the heat maps.

Gene enrichment calculations were performed using the DAVID bioinformatics database tool version 6.8 (https://david.ncifcrf.gov/). The analysis was performed using comparisons of successive time points within the B2 haplotype data to identify sets of genes that were enriched (p-value < 0.05). The B2 haplotype represents the robust macrophage phenotype as characterized by nitric oxide production compared to the B19 haplotype. Subsequently, the gene enrichment was performed on the B2 data. Gene enrichment was determined using three distinct databases: gene ontology biological process, KEGG pathways, and reactome pathways corresponding to S2, S3 and S4 Tables respectively. Because a large number of enrichment annotation terms were produced, a subset of representative highlights from each of these three enrichment analyses was chosen for inclusion in the results. Highlights were selected to provide examples of the biological process annotations, KEGG pathways annotations and reactome annotations.

### PCR validation of target genes

Realtime PCR was performed on a selected number of target genes to validate RNA sequencing results. RNA was taken from macrophages stimulated with IFNγ as described above for 2 and 4 hours, unstimulated samples (0h) served as control. For Realtime RT-PCR, cDNA synthesis was performed using SuperScript III First Strand Synthesis kit (Invitrogen, Waltham, MA), according to manufacturer’s instructions. PCR conditions were as follows: 95°C for 10 min-hot start, 40 cycles of 95°C for 15 sec, 60 or 63°C depending on gene (see primers) for 30 sec according to manufacturer instructions for the Biotool 2x Sybr Green qPCR Mix (Biotool, Houston, Tx). Primer sequences were designed using Primer 3 (ATP6VOC, LITAF, IL18R, TLN-1.) Primer sequences previously published were used for TLR2, TLR4, TLR5, TLR6 and TLR7 [45]. ATP6VOC (annealing 60°C) forward TGTTGTCATGGCGGGTATTA, reverse ACAAATAACCTGGGCTGCTG; LITAF(annealing 60°C) forward ATCCTCACCCCTACCCTGTC, reverse GACGTGTCACGATCATCTGG; IL18R (annealing 63°C) forward CTCTTCGTGCCTCCATTGAT, reverse ACCAAGTTCAACTGGCCAAA; TLN-1(annealing 60°C) forward TCAAGCAGAAGTTGCACACC, reverse GGGAGCCATTAAGGATGTCA. PCR analysis was done using the ΔΔ method with 18s serving as housekeeping gene control. Statistics were done using graphpad software (PRISM version 7), paired t-test, two-tailed.

### Comparison of IFNγ stimulated vs. cytomegalovirus stimulated macrophage gene expression

A total of 179 gene expression measurements were extracted from a published paper describing the fold change in expression levels of genes induced after 4 h exposure to cytomegalovirus [46]. The data was converted to a tab-delimited text file containing the official gene symbol and the reported expression level. The file was loaded into a MySQL relational database and joined to the expression data produced from the B2 and B19 cells. The data was joined on the gene symbol and a set of 54 genes were identified. The fold change for the B2 and B19 expression data was calculated by taking the log-2 (4 h expression / 0 h expression). B2 and B19 genes having expression = 0 for the initial time point were converted to 0.1 to prevent division by zero. Additionally, the fold-change reported for IL6, 280.8, was changed to 35, in order to preserve the scale of the graphs and legibility of the resulting data represented in the histograms. The fold-change in expression for the B-haplotype birds and the published data was plotted using Microsoft Excel.

## Results

### Differential gene expression patterns

A set of 13,618 unique genes from among all mapped sequencing reads was generated from the 4 birds across all 9 time points. Next, we analyzed the expression data to determine the number of genes expressed in each haplotype within each time point. Within the minus 6-day (t-6) time point, representing the time point after plating and adherence of monocytes and the start of differentiation into mature macrophages, 11,785 genes were expressed in the B2 birds while 12,089 were observed in the B19 birds, with 11,216 genes expressed in both. Interestingly, 4770 genes were off in both B19 and B2 haplotypes while just 569 genes exhibited expression in only the B2 chickens and 873 genes were expressed only in the B19 birds.

Similar relationships were detected in each of the remaining eight time points. The t-3 day time point, representing 3 days of differentiation in cell culture, exhibited the greatest expression of genes with a total of 11,429 expressed in both B19 and B2 birds while just 4068 genes lacked evidence of expression in both haplotypes. Also, during the t-3 day time point the greatest number of genes (1118) exhibit evidence of expression in the B2 birds while lacking evidence of expression in the B19 birds. At the t0 time point, after 6 days of differentiation and immediately before stimulation with interferon, 10,975 genes were expressed in both haplotypes while 4547 genes were not expressed in macrophages of either haplotype. Likewise, the 1 h and 2 h time points exhibited 11,349 genes and 10,789, on in both haplotypes, respectively. It is worth noting that the time point with the most genes off in both haplotypes is 16 h with 5238 genes.

**Fig 1:**
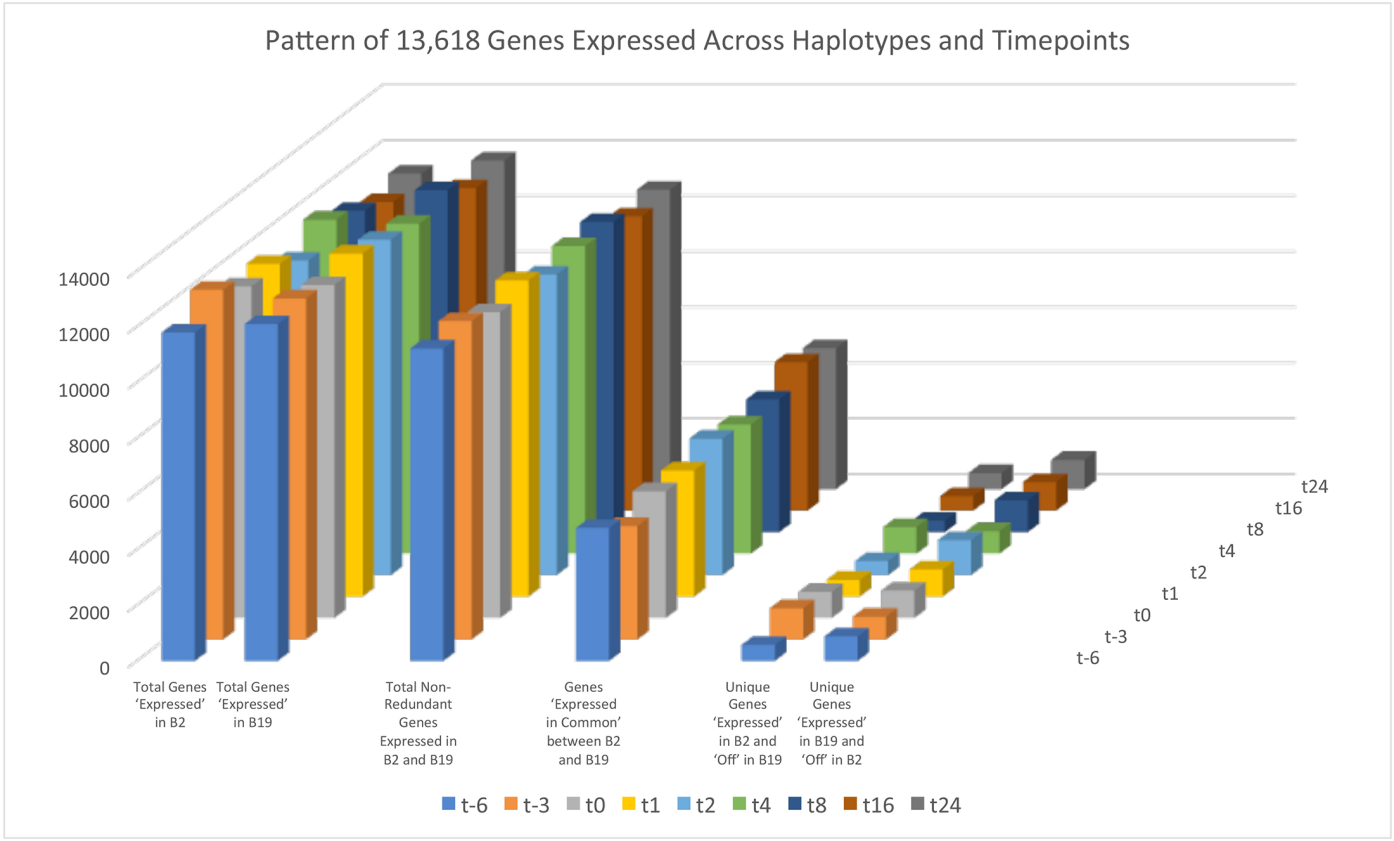
Pattern of 13,618 genes expressed across haplotypes and timepoints. Visual representation of genes within B2 and B19 haplotypes at each of the time points. Figure includes genes expressed in common, genes expressed only in B2, genes expressed only in B19, all genes expressed in B2, all genes expressed in B19, and total non-redundant genes expressed in either B2 or B19 haplotypes.

Overall the data indicates that approximately 10,000 to 11,000 genes are on in both haplotypes at each time point while roughly 4000 to 5300 genes are off in both haplotypes at each time point. The number of genes on in one haplotype, while off in the other haplotype, ranges from about 400 to 1140 depending upon the haplotype and time point (Fig 1)

### Differences in numbers of genes expressed in B19 versus B2 haplotype birds

In order to better understand the cell biology underlying differences in macrophage differentiation and activation between B19 and B2 birds, we searched for genes exhibiting statistically significant differences between different time points within a single B-haplotype haplotype as well within the same time point between haplotypes.

When comparing the expression profiles between the B2 and B19 haplotypes, we identified 210 genes exhibiting differential expression at the t-6 day time point. These genes represent 198 genes with higher expression in the B2 birds and just 12 genes for which expression was greater in the B19. After three days, at the t-3 day time point, thousands of genes exhibited altered expression patterns between the two groups. Surprisingly, 7000 genes showed higher expression in the B19 birds while only 14 genes were expressed at higher levels in the B2 birds.

By t0 hrs, which corresponds to 6 days of monocyte differentiation into macrophages, we observed 955 genes with significant expression patterns between the haplotypes. Of these genes, 544 exhibited greater expression in the B2 haplotype while 411 exhibited higher expression in the B19 haplotype.

Cells were stimulated with IFNγ immediately following RNA collection at the t0 hr time point. At 1 h (t1) post-stimulation 665 genes show evidence of significant patterns of expression between the haplotypes where B19 birds had 109 genes expressed to higher levels while the B19 haplotype was associated with 556 genes having greater expression compared to t0. This pattern of increased expression in the B19 group is reversed by the 2 h time point.

At 2 h after IFNγ treatment, the B2 cells show a global increase in expression for 5989 genes while the B19 cells have just 18 genes on at higher levels than the B2 birds. By 4 hours after stimulation, the B2 birds still exhibit greater expression for 1029 genes while the B19 birds exhibit higher expression for 12 genes. This trend changes by 8 hours after treatment, at which time the slower responding B19 group begin showing increased expression in 797 genes while the B2 cells have greater expression for just 15 genes. By 16 hours after stimulation, only 66 genes are differentially expressed between the two haplotype groups. And, at the 24 hour mark, 406 genes show evidence of statistically significant differences in expression between them with the B2 cells exhibiting greater expression for 339 genes while the B19 cells have higher expression for 67 genes (Table 2).

**Table 2:** Differences in gene expression between B2 and B19.

	Total #	Number Genes	Number Genes
	Significant Genes	Higher in LEFT	Higher in RIGHT
**B2 t-6** versus **B19 t-6**	210	198	12
**B2 t-3** versus **B19 t-3**	7014	14	7000
**B2 t0** versus **B19 t0**	955	544	411
**B2 t1** versus **B19 t1**	665	109	556
**B2 t2** versus **B19 t2**	6007	5989	18
**B2 t4** versus **B19 t4**	1041	1029	12
**B2 t8** versus **B19 t8**	812	15	797
**B2 t16** versus **B19 t16**	66	28	38
**B2 t24** versus **B19 t24**	406	339	67
**B2 t-6** versus **B2 t-3**	6012	5998	14
**B2 t-3** versus **B2 t0**	523	379	144
**B2 t0** versus **B2 t1**	534	339	195
**B2 t1** versus **B2 t2**	6104	6	6098
**B2 t2** versus **B2 t4**	621	391	230
**B2 t4** versus **B2 t8**	6185	6185	0
**B2 t8** versus **B2 t16**	83	39	44
**B2 t16** versus **B2 t24**	0	0	0
**B19 t-6** versus **B19 t-3**	326	14	312
**B19 t-3** versus **B19 t0**	7157	7144	13
**B19 t0** versus **B19 t1**	67	1	66
**B19 t1** versus **B19 t2**	180	159	21
**B19 t2** versus **B19 t4**	1227	63	1164
**B19 4** versus **B19 8**	70	20	50
**B19 8** versus **B19 16**	386	362	24
**B19 16** versus **B19 24**	24	11	13

### Different temporal gene expression in B19 versus B2 haplotype birds

**Fig 2:**
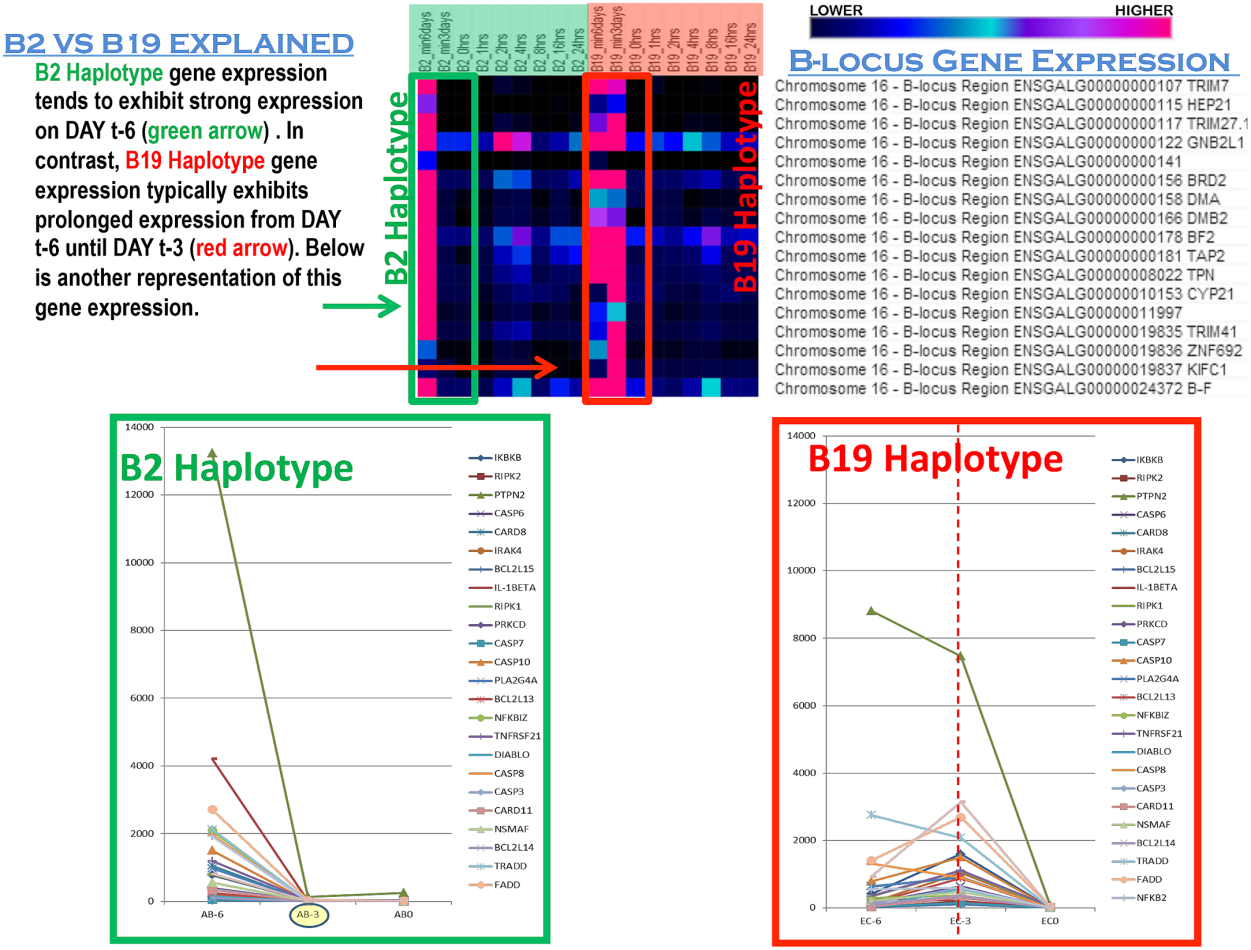
Distinct temporal gene expression patterns in B2 versus B19 monocytes/macrophages. B-locus haplotypes in chickens provide a mechanism for genetically perturbing the cluster of immunologically important genes on chromosome 16 and producing phenotypic variation affecting infectious disease susceptibility and resistance. The heat map allows visualization of gene expression between the two genetically distinct haplotypes. Each row represents a gene within the B-locus (listed on the right) and each column corresponds to a particular time point when cells were collected for RNA sequencing. Black pixels indicate zero gene expression for a particular gene at a specific point in time, and dark blue corresponds to very low expression, while brighter blue indicates the next higher levels. Dark purple represents higher expression levels than blue colors, and pink represents the highest levels of gene expression. Monocytes were obtained from each haplotype of chicken and allowed to differentiate into macrophages in vitro for seven, days beginning on day minus 6 (t-6). RNA was sampled on day t-6, day t-3, and again three days later which is denoted as 0 hours (t0), when IFNγ was initially added to the cultures. On t0, RNA was sampled immediately before stimulation with IFNγ. Subsequent time points correspond to the time following interferon stimulation, in hours (1 hour, 2 hours, 4 hours, 8 hours, 16 hours and 24 hours). As visible on the heat map, there are distinct differences in gene expression between the B2 and B19 cells. The most dramatic difference occurs on day t-6. B2 cells exhibit a rapid burst of gene expression, indicated as a single column of pink on the left most edge of the heat map. In contrast, the B19 cells appear to undergo a much slower and prolonged gene expression program that was not as rapidly down regulated as in genes in the B2 cells. Additional gene expression data for a number of proteins involved in cell growth and apoptosis, is shown in the bottom half of the figure to highlight a similar pattern in gene expression and kinetics. The green border indicates the B2 haplotype expression pattern and the red border corresponds to the B19 expression pattern.

The B2 and B19 haplotype birds represent distinct genetic variation within the B-locus on chromosome 16. Subsequently, patterns of gene expression variation of the genes located within this region were investigated. Among the seventeen genes exhibiting statistically significant differences in expression between the B2 and B19 birds, many displayed divergent gene expression patterns prior to IFNγ stimulation. In the B2 cells, gene expression peaks on day t-6 and expression is effectively inhibited by day t-3. This is not the case in the B19 cells. Rather than reach maximum expression levels in a single day, the B19 cells don’t achieve maximum expression until day t-3 (Fig 2).

For example TRIM7, TRIM27.1, BF2, TPN, and TRIM41 exhibit strong expression on day t-6 in the B2 cells while the same genes exhibited prolonged expression over day t-6 and day t-3 in the B19 cells. Members of the TRIM (tripartite motif) family have been implicated in antiviral immune defense and several are ubiquitin ligases [47, 48]. TPN (Tapasin) is a co-factor for MHC I critical for antigen presentation to cytotoxic T-cells and chickens express the single MHCI locus termed BF-2 which is working with TPN in antigen presentation and it has been shown that there are differences in the selection of high affinity peptides in B19 vs B15 haplotypes [49] highlighting their critical role in immune competence. Additional genes within the B-locus display a similar pattern of pre-stimulatory differences in gene expression between the two different haplotypes, including genes involved in differentiation, cell growth and apoptosis such as PTPN2 (tyrosine protein phosphatase non-receptor2) and NFKB. Gene expression decreases to approximately baseline levels by time point t0 hours.

A second distinction in the gene expression patterns between B19 and B2 cells is that B2 cells exhibited a fairly robust expression at 2 and 4 hours after interferon stimulation. Unlike the B2 haplotype, the B19 haplotype appears incapable of generating such a rapid, robust and coherent gene expression profile. In contrast, the B19 cells generate a delayed, weak and uncoordinated lower level of expression that extends up to 8 hours, and in some cases even 16 hours. Overall, this global pattern of temporally dysregulated gene expression represents a re-occurring theme with the B19 monocytes and macrophages.

The divergent timing of gene expression observed in the B-locus genes is mirrored in many other genes as well, including members of the TLR signaling pathway, cellular mediators of apoptosis and cell survival, and components of cytokine signaling.

### B2 and B19 display different patterns of gene expression during differentiation

**Fig 3:**
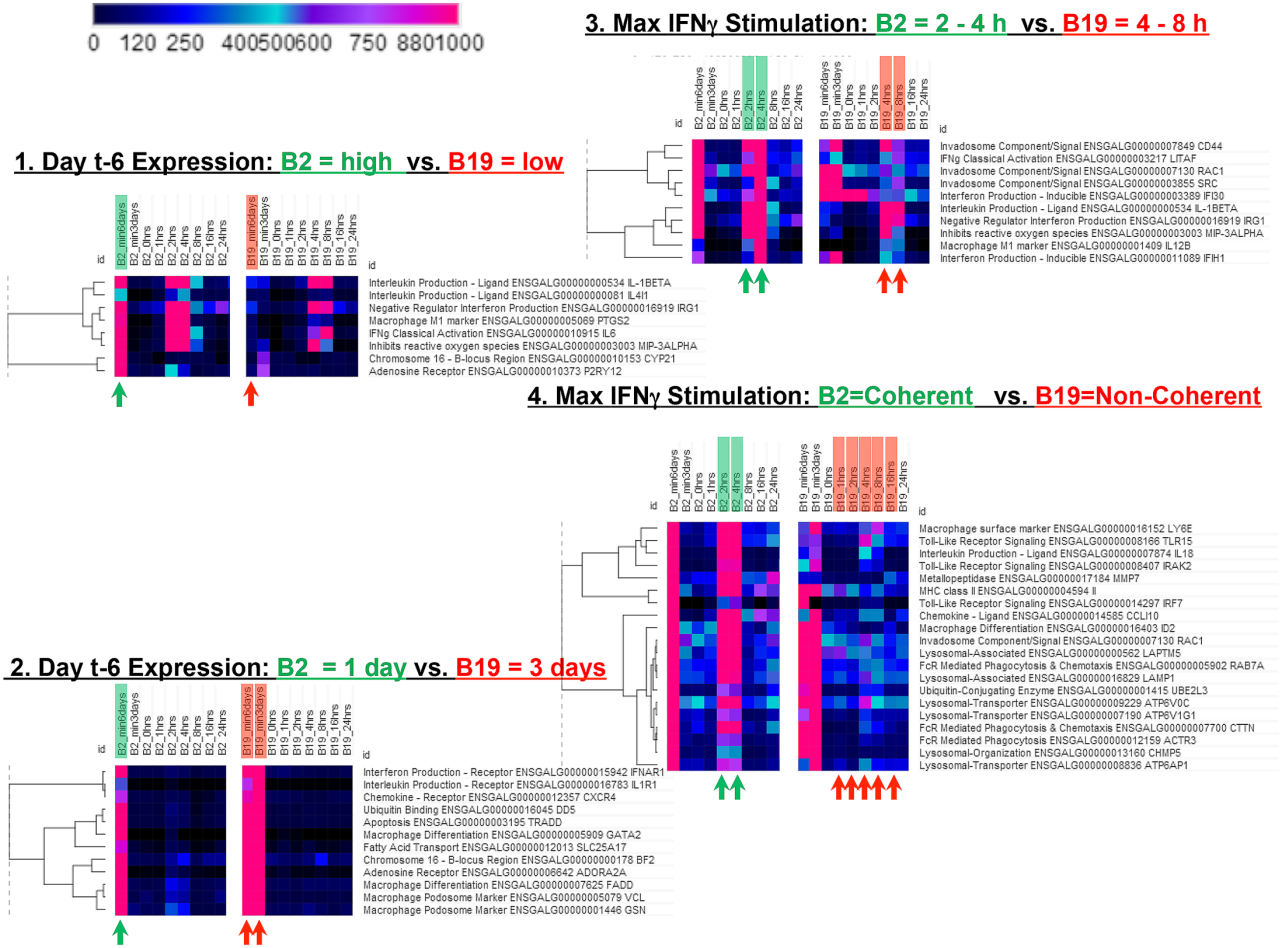
Examples of divergent gene expression patterns observed in B2 and B19 haplotype macrophages. Four distinct patterns were identified as representative of the types of divergent gene expression that re-occur across many genes involved in macrophage differentiation, activation and function in B2 versus B19 macrophages. 1. **Day t-6: B2 high vs B19 low**. This divergent pattern exhibits strong expression of genes on day -6 in the B2 birds while relatively low levels of expression are observed in the B19 birds at the same time point. Genes of interest include an adenosine receptor (P2RY12) 2. **Day t-6: B2 = 1 day vs. B19 = 3 days.** This example of divergent patterns is the single peak of day t-6 gene expression in the B2 haplotype cells compared to the prolonged multiple day expression until day t-3 in the B19 haplotype cells. Genes of interest include macrophage differentiation gene GATA, adenosine receptor A2A and macrophage podosome markers VCL and GSN. **3. Maximum** **IFN**γ **Stimulation of B2 at 2–4 h versus 4–8 h in B19 macrophages.** Another interesting divergent gene expression pattern observed between the two haplotypes occurs after stimulation by IFNγ. There is a four-hour difference in peak expression timing for a large number of induced genes. In the B2 haplotype macrophages, the peak expression occurs between 2 and 4 hours, while in the B19 macrophages, the peak expression occurs between 4 and 8 hours. **4. Maximum** **IFN**γ **Stimulation: B2 = Coherent vs. B19 = Non-Coherent** Another discernable difference in post-stimulatory induction of genes between the B2 macrophages compared to the B19 macrophages is one of coherence. Specifically, there are a number of genes for which the B2 macrophages are able to rapidly turn on and reach relatively high levels of expression within 2 to 4 hours of IFNγ stimulation. In contrast, these same genes fail to exhibit a coherent peak of expression, even after 4 to 8 hours, in the B19 cells. Instead, they exhibit a dispersed “smear” of gene expression extending from approximately 1 hour after stimulation to 16 hours post-stimulation.

The global dysregulation of gene expression among 700 genes at the t-3 day time point, as well as the expression pattern of 6000 genes exhibiting altered expression led us to explore the pattern of gene expression changes within each haplotype group over all of the time points. At the onset of the study, the B2 cells were actively expressing a diverse set of genes, however by the day t-3, most of those genes displayed reduced expression in the B2 group. Even so, the B19 haplotype cells continue to express these 7000 genes at higher levels than the B2 birds. After stimulation, B2 macrophages again show different patterns of expression compared to B19 cells in regards to timing of peak expression and coherence of expression. Four distinct patterns of divergent gene expression were identified between the B2 haplotype birds and the B19 haplotype birds (Fig 3).

The first interesting divergent pattern shows strong gene expression on day t-6 in the B2 birds while relatively low levels of expression are observed in the B19 birds on the same day. This pattern is of interest because it represents a group of genes that are differentially regulated at the onset of the experimental time course. Specifically, these genes include the macrophage M1 marker PTGS2, as well as the B-locus gene cyp21. Other genes exhibiting this pattern include secreted interleukin ligands IL-1β, IL4I1, and IL6, along with genes associated with inhibition of cellular processes including IRG1 and MIP-3α. Interestingly, the adenosine receptor also displays this pattern of expression. These genes may represent initial modulators of divergent monocyte to macrophage differentiation between the B2 and B19 cells.

The second example of divergent expression patterns is the single peak of day t-6 expression in the B2 haplotype cells compared to the prolonged multiple day expression in the B19 haplotype cells. Some of these genes are macrophage differentiation mediators, like GATA2 [50], and FADD, while others are macrophage podosome (primary matrix structure) markers, including VCL and GSN. Other genes exhibiting this divergent expression pattern include chemokine receptors, like CxCR4, fatty acid transport, such as SLC25A17, and ubiquitin related factors, like DD5, which is associated with proteasomal degradation of gene products.

Additional interesting divergent gene expression patterns were observed between the two haplotypes occurring after stimulation by IFNγ (Fig 3). A notable difference in post-stimulatory induction of gene expression is a four-hour difference in peak expression timing for a large number of induced genes. In the B2 haplotype macrophages, the peak expression occurs between 2 and 4 hours, while in the B19 macrophages, the peak expression occurs between 4 and 8 hours. Some of the most noticeable genes exhibiting this divergent gene expression pattern include LITAF, IL-1β, IL12, and IFIH1, genes involved in macrophage signaling and M1 macrophage polarization [26]. Additionally, a number of genes implicated in invadosome assembly and function also exhibit this temporally displaced pattern of induction such as CD44, RAC1, and SRC.

Another discernable difference in post-stimulatory induction of genes between the B2 macrophages compared to the B19 macrophages is one of coherence (Fig 3). Specifically, there are a number of genes for which the B2 macrophages are able to rapidly turn on and reach relatively high levels of expression within 2 to 4 hours of IFNγ stimulation. In contrast, these same genes fail to exhibit a coherent peak of expression, even after 4 to 8 hours, in the B19 cells. Instead, they exhibit a dispersed “smear” of gene expression extending from approximately 1 hour after stimulation to 16 hours post-stimulation. Some of the most represented genes exhibiting this divergent pattern of expression include molecules involved in lysosome function and phagocytosis. CTTN and ACTR3, genes implicated in FcR mediated phagocytosis, along with lysosomal-associated molecules, like LAPTM5 and LAMP1, as well as the lysosomal transporter molecules ATP6AP1, ATP6V1G1 and ATP6V0C, exhibit this non-coherent pattern of expression in the B19 macrophages.

In contrast, immediately following stimulation, the B2 cells rapidly induce expression of roughly 6000 genes by the 2 h following stimulation; while, at the same time, the cells derived from the B19 birds show no signs of induction among these genes until after 4 h. It is interesting to note that while the B2 birds show a statistically significant increase in expression for 6100 genes between 1 h and 2 h, the B19 cells exhibit increased expression for just 66 genes at this time point. The largest wave of increased gene expression occurs in the B19 cells during the transition from 2 h to 4 h post stimulation, when 1164 genes increase significantly over this time period.

At the transition between 8 h and 16 h, the B2 haplotype group only exhibits differences in expression for 83 genes, with 44 having higher expression at the 16 h time point. Yet, the B19 cells show differences in 386 genes during this same period, but interestingly, 356 of these genes exhibit decreased expression during this same time interval. Taken together, these results suggest that a global disruption of temporal gene expression underlies the observed differences in differentiation, activation and nitric oxide production from macrophages derived from the two different MHC haplotypes.

### RT-PCR of B2 and B19 haplotype cells following IFNγ stimulation

**Fig 4:**
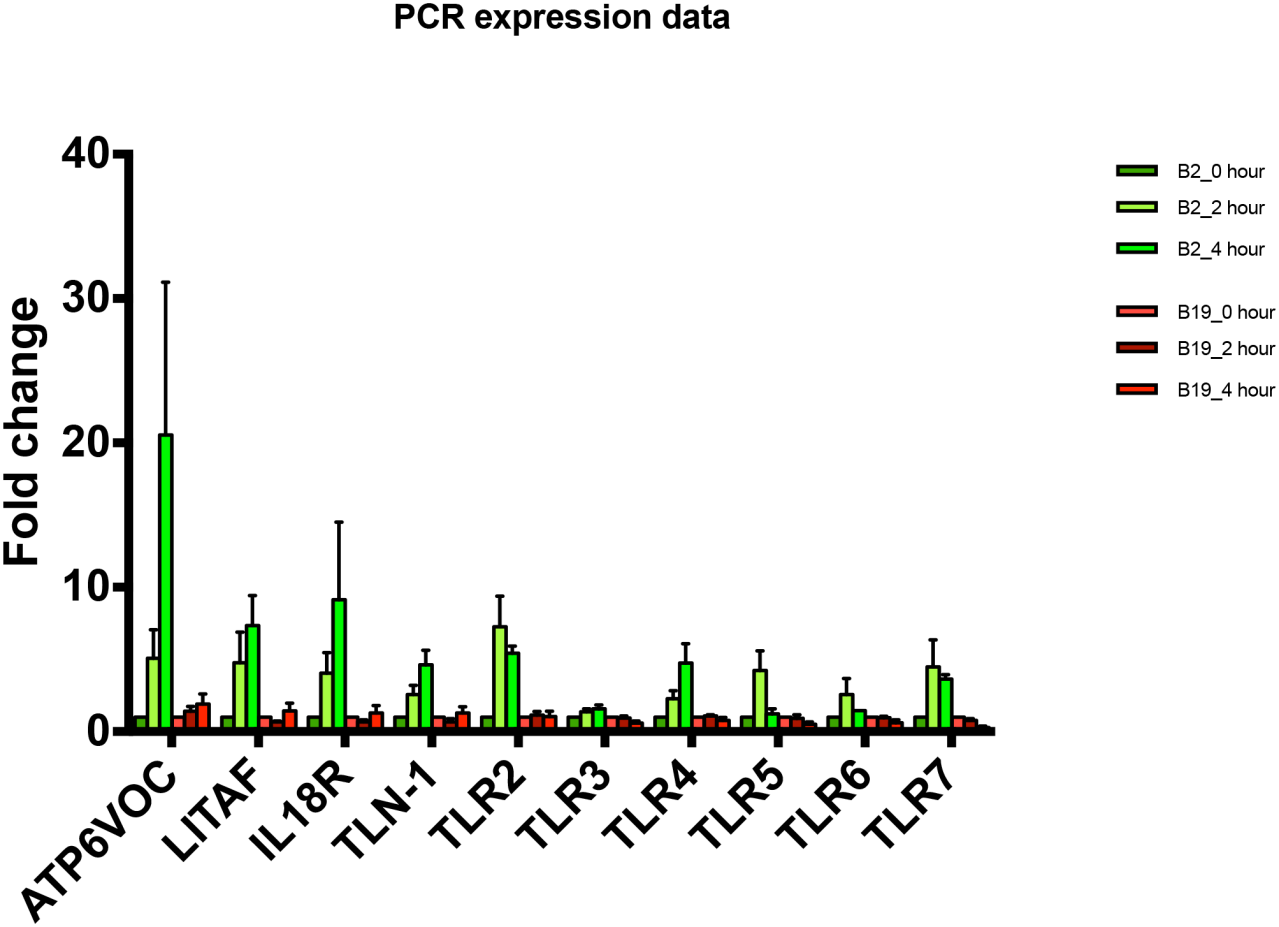
RT-PCR validation of transcripts identified as significantly expressed in RNA sequencing data. Gene expression for ATP6V0C, LITAF, IL18R, TLN-1, TLR2, TLR3, TLR4, TLR5, TLR6, and TLR7 was assessed in B2 and B19 monocytes/macrophages following stimulation with IFNγ. Expression was measured at 0 hours, 2 hours and 4 hours. Expression for transcripts in B2 cells are shown in green and expression for transcripts are shown in red. Standard error is shown for each value. Values were considered statistically significant with p<0.05.

Gene expression was measured in separate samples of B2 and B19 cells following stimulation with IFNγ. Change in expression was assessed at 2 hours and 4 hours post stimulation. ATP6V0C exhibited the greatest induction of all genes assayed, showing an increased expression in the B2 cells at 4 hours that was 20 times the initial expression at 0-hours. Expression of ATP6VOC was dramatically less in the B19 birds. Similarly, IL18R exhibited greater than 9 times the initial expression in the B2 cells at 4 hours compared to the B19 cells which exhibited less than 2 times the initial expression at 0-hours. LITAF and TLR2 exhibited more than 7 times the expression at 4 hours in the B2 macrophages, while TLN-1, TLR-5, TLR-6 and TLR-7 exhibited greater than 4 times the initial expression in the B2 macrophages. In contrast, the B19 macrophages failed to exhibit comparable induction of these genes (Fig 4).

### IFNγ stimulated vs. cytomegalovirus stimulated macrophage gene expression

**Fig 5:**
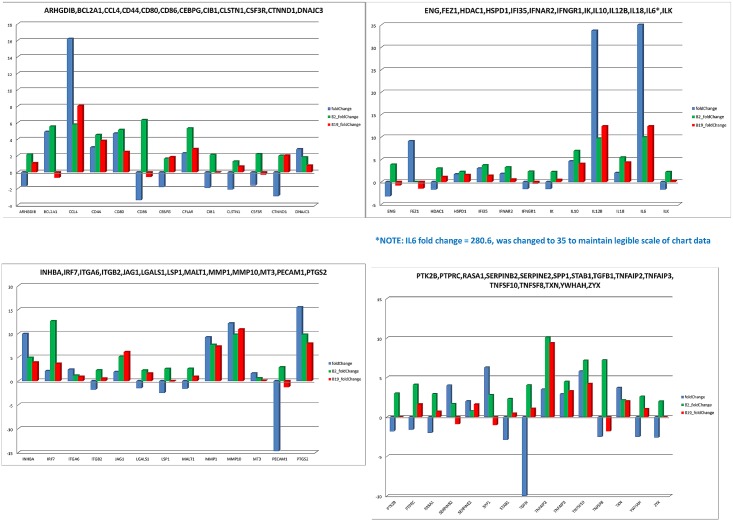
IFNγ stimulated vs. cytomegalovirus stimulated macrophage gene expression. 54 genes, for which gene expression changes were previously described following cytomegalovirus stimulation were used as comparisons for the corresponding genes in the B2 and B19 haplotype birds. A total of 25 published genes exhibited decreases in expression following cytomegalovirus stimulation while 29 genes exhibited increased expression following stimulation. All but one gene (FEZ1) in the B2 cells exhibited increased expression following IFNγ stimulation. In contrast, ten genes displayed decreased expression in the B19 cells. Of the ten exhibiting fold-change < 0 in the B19 cells, seven exhibited decreased expression in the cytomegalovirus stimulated cells. Twenty-eight genes (52%) expressed in the B2 cells matched the direction of the fold change reported in the published data while 33 genes (61%) corresponded between the B19 cells and the published data. Of the ten published genes reported as having greater than at least 5-fold increased expression, 90% of the B2 genes exhibited fold-change in the same direction.

In addition to the RT-PCR validation of gene expression, 54 genes, for which gene expression changes were described following cytomegalovirus stimulation were used as comparisons for the corresponding genes in the B2 and b19 haplotype birds (Fig 5). A total of 25 published genes exhibited decreases in expression following cytomegalovirus stimulation while 29 genes exhibited increased expression following stimulation. Interestingly, all but one gene (FEZ1) in the B2 cells exhibited increased expression following IFNγ stimulation. In contrast, ten genes displayed decreased expression in the B19 cells. Of the ten exhibiting fold-change < 0 in the B19 cells, 70% also exhibited decreased expression in the cytomegalovirus stimulated cells. In total, 28 genes (52%) expressed in the B2 cells matched the direction of the fold change reported in the published data while 33 genes (61%) corresponded between the B19 cells and the published data. Of the ten published genes reported as having greater than 5-fold increased expression, 90% of the B2 genes exhibited fold-change in the same direction. Overall, this data, in conjunction with the RT-PCR data, provides a comprehensive set of validation data providing evidence that the B2 and B19 gene expression data is reproducible and similar to expression patterns observed in cells stimulated towards macrophage activation pathway.

### Divergent non-coding RNA expression in B2 and B19 macrophages

**Fig 6:**
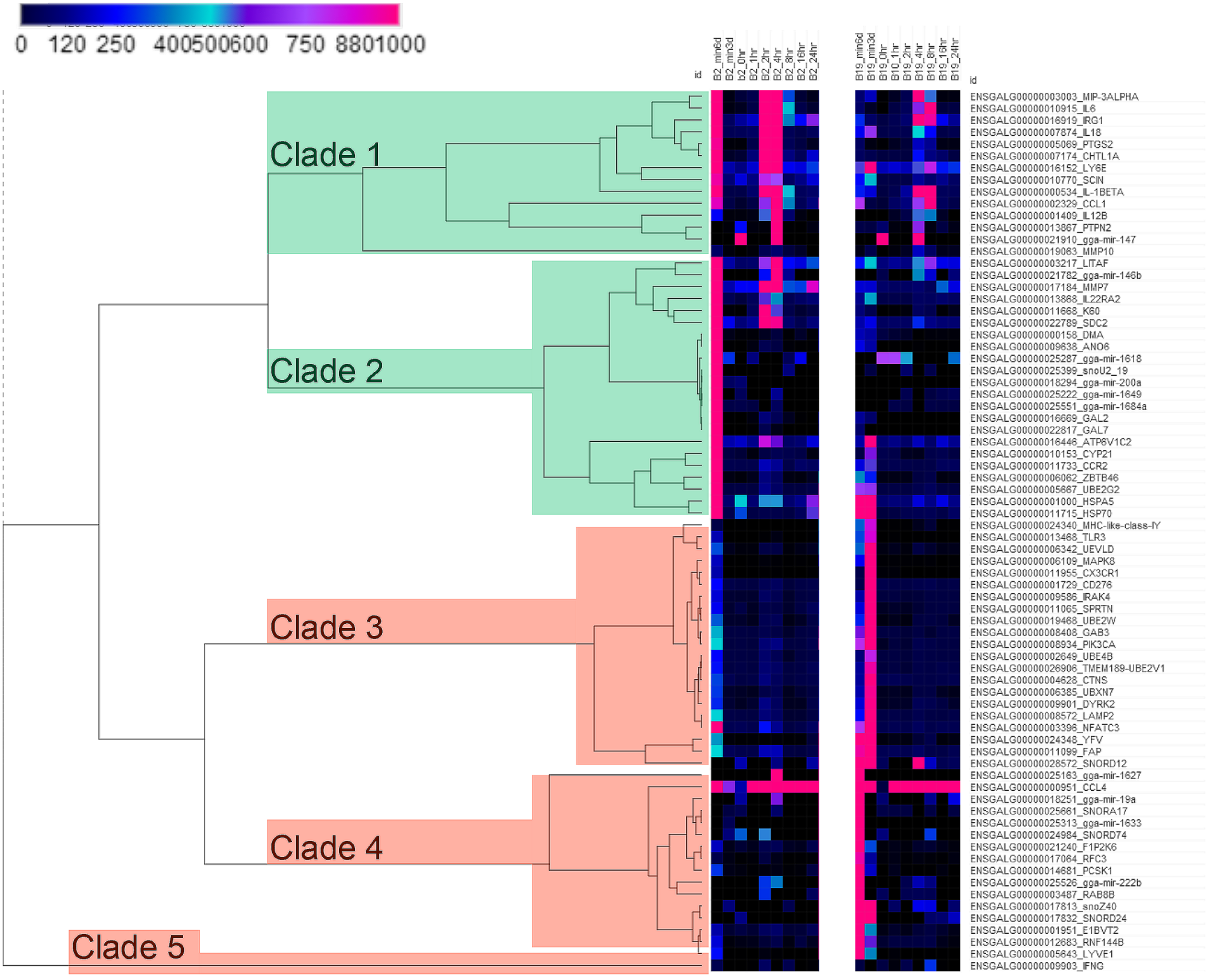
Identification of divergent gene expression patterns between B2 and B19 macrophages. Visualization of divergent gene expression patterns between the B2 and B19 haplotypes. A subset of genes exhibiting divergent gene expression were identified and visualized in heat following hierarchical clustering of the genes (rows), but not the time points (columns). The genes cluster into four major clades (clade1, clade2, clade3, and clade4) with a singleton gene (labelled clade 5). Among these genes, represented in clade1 and clade2, are a number of miRNAs exhibiting strong expression in B2 cells (mir-147, mir-146b, mir-1618, mir-200a, mir-1649, and mir-1648a) compared to the B19 samples. Likewise, miRNAs contained in clade3 and clade4 exhibit greater expression in B19 cells (mir-1627, mir-222b, mir-1633, and mir-19a). Additionally, a number of small nucleolar RNAs (snoRNAs) exhibit similarly dichotomous gene expression patterns (clade4) such that SNORd24, snoZ40, SNORD74, SNORA17, and SNORD12 exhibit substantially higher levels of expression in B19 cells on day -6 compared to B2 cells while B2 cells express such as snoU2_19 (clade2).

Visualization of gene expression via heat maps facilitated the identification of distinct expression patterns between the B2 and B19 haplotypes. Because any initial differences in gene expression existing 6 days before IFNγ stimulation represent candidates responsible for the observed phenotypic differences between the two haplotypes. Genes exhibiting divergent gene expression patterns between B2 and B19 birds on day -6 were identified (Fig 6). The genes cluster into four major clades (clade1, clade2, clade3, and clade4 with a singleton labelled clade 5). Among these genes, represented in clade1 and clade2, are a number of miRNAs exhibiting strong expression in B2 cells (mir-147, mir-146b, mir-1618, mir-200a, mir-1649, and mir-1648a) compared to the B19 samples. Likewise, miRNAs contained in clade3 and clade4 exhibit greater expression in B19 cells (mir-1627, mir-222b, mir-1633, and mir-19a).

A number of small nucleolar RNAs (snoRNAs) exhibit similarly dichotomous gene expression patterns (clade4). For example, SNORd24, snoZ40, SNORD74, SNORA17, and SNORD12 exhibit substantially higher levels of expression in B19 cells on day -6 compared to B2 cells (Fig 6). However, B2 cells also express snoRNAs exhibiting divergent expression patterns between the two haplotypes, such as snoU2_19 (clade2).

In addition to non-coding RNAs, divergent expression patterns are also observed with protein-coding RNAs (Fig 6). For example, clade1 contains IL6, IL18, IL-1β, CCL1, PTPN2 and MMP10, which exhibit higher initial expression on day -6 in the B2 birds. In contrast, the protein-coding genes LAMP2, UBXN7, UBE4B, PK3CA, UBE2W, and CX3CR1, in clade3, exhibit higher initial expression patterns in B19 birds. Clade5 contains the single gene IFNγ, which exhibits relatively low expression early in both B2 and B19 cells, but following stimulation rises to a higher level at 8 hours in the B19 birds.

Considering the diverse expression patterns discovered and the results indicating the involvement of non-coding RNAs, further results including divergent non-coding RNA expression will be described in more detail in further publications.

### Gene enrichment analysis

Enrichment analysis of genes exhibiting statistically significant differences in expression between time points and/or haplotypes (Table 3, S2, S3 and S4 Tables) was performed using gene ontology and both KEGG and reactome pathways. The results of the gene enrichment provided a high-resolution perspective of the functional role of mRNA sequenced within the B2 cells across the experimental time points.

**Table 3:** Gene enrichment analysis—Highlights from gene ontology, KEGG pathways, reactome pathways.

Sample Comparison	Gene Set	Term	Description	Gene Count	P-Value
B2 t-6 vs. B2 t-3	Down	GO:0006360	transcription from RNA polymerase I promoter	12	8.08E-05
B2 t-6 vs. B2 t-3	Down	GO:0000398	mRNA splicing, via spliceosome	33	8.85E-05
B2 t-6 vs. B2 t-3	Down	GO:0043966	histone H3 acetylation	20	4.18E-04
B2 t-6 vs. B2 t-3	Down	GO:0008333	endosome to lysosome transport	17	0.001611612
B2 t-6 vs. B2 t-3	Down	GO:0008033	tRNA processing	12	0.002902509
B2 t-6 vs. B2 t-3	Down	GO:0031338	regulation of vesicle fusion	15	0.005935057
B2 t-6 vs. B2 t-3	Down	GO:0006378	mRNA polyadenylation	11	0.006175743
B2 t-6 vs. B2 t-3	Down	GO:0000387	spliceosomal snRNP assembly	13	0.006325454
B2 t-6 vs. B2 t-3	Down	GO:0006397	mRNA processing	27	0.010374791
B2 t-6 vs. B2 t-3	Down	GO:0006886	intracellular protein transport	61	0.012613014
B2 t-6 vs. B2 t-3	Down	GO:0043123	positive regulation of I-kappaB kinase/NF-kappaB signaling	42	0.013554129
B2 t-6 vs. B2 t-3	Down	GO:0016050	vesicle organization	11	0.023348647
B2 t-6 vs. B2 t-3	Down	GO:0030968	endoplasmic reticulum unfolded protein response	16	0.023521606
B2 t-6 vs. B2 t-3	Down	GO:0045022	early endosome to late endosome transport	10	0.024838372
B2 t-6 vs. B2 t-3	Down	GO:0045292	mRNA cis splicing, via spliceosome	7	0.025727402
B2 t-6 vs. B2 t-3	Down	GO:0016226	iron-sulfur cluster assembly	9	0.047036045
B2 t-6 vs. B2 t-3	Down	GO:0007032	endosome organization	13	0.048431064
B2 t-6 vs. B2 t-3	Down	GO:0032088	negative regulation of NF-kappaB transcription factor activity	16	0.048807155
B2 t-6 vs. B2 t-3	Down	gga03020	RNA polymerase	17	8.84E-05
B2 t-6 vs. B2 t-3	Down	gga03018	RNA degradation	38	6.00E-04
B2 t-6 vs. B2 t-3	Down	gga01100	Metabolic pathways	432	0.001331836
B2 t-6 vs. B2 t-3	Down	gga03008	Ribosome biogenesis in eukaryotes	37	0.002017081
B2 t-6 vs. B2 t-3	Down	gga03040	Spliceosome	54	0.002180456
B2 t-6 vs. B2 t-3	Down	gga00190	Oxidative phosphorylation	57	0.004584223
B2 t-6 vs. B2 t-3	Down	gga03022	Basal transcription factors	21	0.021845672
B2 t-6 vs. B2 t-3	Down	gga03060	Protein export	14	0.025867798
B2 t-6 vs. B2 t-3	Down	gga03015	mRNA surveillance pathway	34	0.034772067
B2 t-6 vs B2 t-3	Down	R-GGA-5419276	Mitochondrial translation termination	41	4.06E-10
B2 t-6 vs B2 t-3	Down	R-GGA-5389840	Mitochondrial translation elongation	39	5.57E-09
B2 t-6 vs B2 t-3	Down	R-GGA-73779	RNA Polymerase II Transcription Pre-Initiation And Promoter Opening	24	5.04E-06
B2 t-6 vs B2 t-3	Down	R-GGA-75953	RNA Polymerase II Transcription Initiation	24	5.04E-06
B2 t-6 vs B2 t-3	Down	R-GGA-76042	RNA Polymerase II Transcription Initiation And Promoter Clearance	24	5.04E-06
B2 t-6 vs B2 t-3	Down	R-GGA-674695	RNA Polymerase II Pre-transcription Events	34	5.58E-06
B2 t-6 vs B2 t-3	Down	R-GGA-72086	mRNA Capping	19	1.15E-05
B2 t-6 vs B2 t-3	Down	R-GGA-75955	RNA Polymerase II Transcription Elongation	24	1.38E-04
B2 t-6 vs B2 t-3	Down	R-GGA-72165	mRNA Splicing—Minor Pathway	26	2.60E-04
B2 t-6 vs B2 t-3	Down	R-GGA-72163	mRNA Splicing—Major Pathway	51	6.17E-04
B2 t-6 vs B2 t-3	Down	R-GGA-983168	Antigen processing: Ubiquitination & Proteasome degradation	32	0.001262633
B2 t-6 vs B2 t-3	Down	R-GGA-1834949	Cytosolic sensors of pathogen-associated DNA	12	0.001290237
B2 t-6 vs B2 t-3	Down	R-GGA-611105	Respiratory electron transport	24	0.002379624
B2 t-6 vs B2 t-3	Down	R-GGA-1855183	Synthesis of IP2, IP, and Ins in the cytosol	7	0.009896133
B2 t-6 vs B2 t-3	Down	R-GGA-180292	GAB1 signalosome	8	0.028654133
B2 t-6 vs B2 t-3	Down	R-GGA-189451	Heme biosynthesis	7	0.027634144
B2 t-3 vs. B2 t0	Down	GO:0007059	chromosome segregation	8	1.87E-05
B2 t-3 vs. B2 t0	Down	GO:0007067	mitotic nuclear division	7	0.002285326
B2 t-3 vs. B2 t0	Down	GO:0007018	microtubule-based movement	6	0.003424586
B2 t-3 vs. B2 t0	Down	GO:0000281	mitotic cytokinesis	4	0.004035208
B2 t-3 vs. B2 t0	Down	GO:0008152	metabolic process	6	0.01138645
B2 t-3 vs. B2 t0	Down	GO:0006281	DNA repair	7	0.021563304
B2 t-3 vs. B2 t0	Down	GO:0045671	negative regulation of osteoclast differentiation	3	0.02255698
B2 t-3 vs. B2 t0	Down	GO:0051301	cell division	6	0.030134621
B2 t-3 vs. B2 t0	Down	gga04110	Cell cycle	14	5.77E-06
B2 t-3 vs. B2 t0	Down	gga03030	DNA replication	7	1.16E-04
B2 t-3 vs. B2 t0	Down	gga03430	Mismatch repair	5	0.00172114
B2 t-3 vs. B2 t0	Down	gga00240	Pyrimidine metabolism	9	0.002460978
B2 t-3 vs. B2 t0	Down	gga04630	Jak-STAT signaling pathway	8	0.034070609
B2 t-3 vs. B2 t0	Down	R-GGA-5663220	RHO GTPases Activate Formins	14	1.85E-07
B2 t-3 vs. B2 t0	Down	R-GGA-2500257	Resolution of Sister Chromatid Cohesion	14	2.19E-07
B2 t-3 vs. B2 t0	Down	R-GGA-2467813	Separation of Sister Chromatids	14	1.41E-06
B2 t-3 vs. B2 t0	Down	R-GGA-69205	G1/S-Specific Transcription	5	2.53E-04
B2 t-3 vs. B2 t0	Down	R-GGA-113510	E2F mediated regulation of DNA replication	5	2.53E-04
B2 t-3 vs. B2 t0	Down	R-GGA-983189	Kinesins	4	0.001487779
B2 t-3 vs. B2 t0	Down	R-GGA-156582	Acetylation	3	0.002846986
B2 t-3 vs. B2 t0	Down	R-GGA-5358565	Mismatch repair (MMR) directed by MSH2:MSH6 (MutSalpha)	4	0.003044778
B2 t-3 vs. B2 t0	Down	R-GGA-5651801	PCNA-Dependent Long Patch Base Excision Repair	4	0.00409159
B2 t-3 vs. B2 t0	Down	R-GGA-512988	Interleukin-3, 5 and GM-CSF signaling	4	0.006774634
B2 t-3 vs. B2 t0	Down	R-GGA-912526	Interleukin receptor SHC signaling	3	0.030200217
B2 t-3 vs. B2 t0	Up	GO:0071353	cellular response to interleukin-4	4	1.26E-04
B2 t-3 vs. B2 t0	Up	GO:0006564	L-serine biosynthetic process	2	0.025467339
B2 t-3 vs. B2 t0	Up	GO:0006166	purine ribonucleoside salvage	2	0.033813353
B2 t-3 vs. B2 t0	Up	GO:0006366	transcription from RNA polymerase II promoter	4	0.047682647
B2 t-3 vs. B2 t0	Up	gga04141	Protein processing in endoplasmic reticulum	7	0.001678292
B2 t-3 vs. B2 t0	Up	gga01230	Biosynthesis of amino acids	4	0.015296859
B2 t-3 vs. B2 t0	Up	gga01100	Metabolic pathways	16	0.036140022
B2 t-3 vs. B2 t0	Up	gga00260	Glycine, serine and threonine metabolism	3	0.03864845
B2 t-3 vs. B2 t0	Up	R-GGA-977347	Serine biosynthesis	2	0.023430989
B2 t-3 vs. B2 t0	Up	R-GGA-433692	Proton-coupled monocarboxylate transport	2	0.046329225
B2 t0 vs B2 t1	Down	GO:0006412	translation	17	2.85E-08
B2 t0 vs B2 t1	Down	GO:0042149	cellular response to glucose starvation	6	3.11E-05
B2 t0 vs B2 t1	Down	GO:0006457	protein folding	9	4.18E-04
B2 t0 vs B2 t1	Down	GO:0030968	endoplasmic reticulum unfolded protein response	4	0.023705124
B2 t0 vs B2 t1	Down	GO:0030970	retrograde protein transport, ER to cytosol	3	0.02551737
B2 t0 vs. B2 t1	Down	gga03010	Ribosome	21	7.21E-11
B2 t0 vs. B2 t1	Down	gga04141	Protein processing in endoplasmic reticulum	20	2.24E-08
B2 t0 vs. B2 t1	Down	gga00970	Aminoacyl-tRNA biosynthesis	7	0.001164589
B2 t0 vs. B2 t1	Down	gga00330	Arginine and proline metabolism	6	0.006099731
B2 t0 vs. B2 t1	Down	R-GGA-1799339	SRP-dependent cotranslational protein targeting to membrane	15	4.23E-11
B2 t0 vs. B2 t1	Down	R-GGA-72706	GTP hydrolysis and joining of the 60S ribosomal subunit	14	2.46E-10
B2 t0 vs. B2 t1	Down	R-GGA-975956	Nonsense Mediated Decay (NMD) independent of the Exon Junction Complex (EJC)	14	6.02E-10
B2 t0 vs. B2 t1	Down	R-GGA-975957	Nonsense Mediated Decay (NMD) enhanced by the Exon Junction Complex (EJC)	14	5.10E-09
B2 t0 vs. B2 t1	Down	R-GGA-72695	Formation of the ternary complex, and subsequently, the 43S complex	9	7.35E-07
B2 t0 vs B2 t1	Up	GO:0006954	inflammatory response	11	6.00E-06
B2 t0 vs B2 t1	Up	GO:0051607	defense response to virus	6	6.95E-04
B2 t0 vs B2 t1	Up	GO:0002224	toll-like receptor signaling pathway	4	0.002646723
B2 t0 vs B2 t1	Up	GO:0060326	cell chemotaxis	4	0.006725072
B2 t0 vs B2 t1	Up	GO:0007596	blood coagulation	4	0.012276628
B2 t0 vs B2 t1	Up	GO:0002755	MyD88-dependent toll-like receptor signaling pathway	3	0.012708351
B2 t0 vs B2 t1	Up	GO:0071222	cellular response to lipopolysaccharide	4	0.013230968
B2 t0 vs B2 t1	Up	GO:0007052	mitotic spindle organization	3	0.014697099
B2 t0 vs B2 t1	Up	GO:0006955	immune response	6	0.015620183
B2 t0 vs B2 t1	Up	GO:0002548	monocyte chemotaxis	3	0.019044739
B2 t0 vs B2 t1	Up	GO:0009263	deoxyribonucleotide biosynthetic process	2	0.03982406
B2 t0 vs. B2 t1	Up	gga04620	Toll-like receptor signaling pathway	7	0.001706356
B2 t0 vs. B2 t1	Up	gga04630	Jak-STAT signaling pathway	7	0.008611879
B2 t0 vs. B2 t1	Up	gga04514	Cell adhesion molecules (CAMs)	6	0.026832539
B2 t0 vs. B2 t1	Up	gga04068	FoxO signaling pathway	6	0.038420138
B2 t0 vs. B2 t1	Up	gga05164	Influenza A	6	0.04852434
B2 t0 vs. B2 t1	Up	gga00240	Pyrimidine metabolism	5	0.049377943
B2 t0 vs. B2 t1	Down	R-GGA-1799339	SRP-dependent cotranslational protein targeting to membrane	15	4.23E-11
B2 t0 vs. B2 t1	Down	R-GGA-72706	GTP hydrolysis and joining of the 60S ribosomal subunit	14	2.46E-10
B2 t0 vs. B2 t1	Down	R-GGA-975956	Nonsense Mediated Decay (NMD) independent of the Exon Junction Complex (EJC)	14	6.02E-10
B2 t0 vs. B2 t1	Down	R-GGA-975957	Nonsense Mediated Decay (NMD) enhanced by the Exon Junction Complex (EJC)	14	5.10E-09
B2 t0 vs. B2 t1	Down	R-GGA-72695	Formation of the ternary complex, and subsequently, the 43S complex	9	7.35E-07
B2 t0 vs. B2 t1	Down	R-GGA-72702	Ribosomal scanning and start codon recognition	9	1.44E-06
B2 t0 vs. B2 t1	Down	R-GGA-156590	Glutathione conjugation	3	0.020307126
B2 t0 vs. B2 t1	Down	R-GGA-5673000	RAF activation	3	0.020307126
B2 t0 vs. B2 t1	Down	R-GGA-70614	Amino acid synthesis and interconversion (transamination)	3	0.033683435
B2 t1 vs. B2 t2	Up	GO:0006511	ubiquitin-dependent protein catabolic process	54	5.01E-07
B2 t1 vs. B2 t2	Up	GO:0006886	intracellular protein transport	78	7.15E-06
B2 t1 vs. B2 t2	Up	GO:0006888	ER to Golgi vesicle-mediated transport	31	1.65E-05
B2 t1 vs. B2 t2	Up	GO:0000398	mRNA splicing, via spliceosome	35	8.42E-05
B2 t1 vs. B2 t2	Up	GO:0050821	protein stabilization	42	9.33E-05
B2 t1 vs. B2 t2	Up	GO:0045454	cell redox homeostasis	33	1.41E-04
B2 t1 vs. B2 t2	Up	GO:0007030	Golgi organization	33	6.86E-04
B2 t1 vs. B2 t2	Up	GO:0043001	Golgi to plasma membrane protein transport	13	0.001353221
B2 t1 vs. B2 t2	Up	GO:0008333	endosome to lysosome transport	18	0.001409269
B2 t1 vs. B2 t2	Up	GO:0006338	chromatin remodeling	23	0.005243778
B2 t1 vs. B2 t2	Up	GO:0019827	stem cell population maintenance	21	0.005362005
B2 t1 vs. B2 t2	Up	GO:0000920	cell separation after cytokinesis	11	0.005858508
B2 t1 vs. B2 t2	Up	GO:0071353	cellular response to interleukin-4	10	0.011871699
B2 t1 vs. B2 t2	Up	GO:0016050	vesicle organization	12	0.014441064
B2 t1 vs. B2 t2	Up	GO:0043966	histone H3 acetylation	18	0.014875667
B2 t1 vs. B2 t2	Up	GO:0000381	regulation of alternative mRNA splicing, via spliceosome	14	0.015404037
B2 t1 vs. B2 t2	Up	GO:0031338	regulation of vesicle fusion	15	0.015497515
B2 t1 vs. B2 t2	Up	GO:0034067	protein localization to Golgi apparatus	7	0.015640559
B2 t1 vs. B2 t2	Up	GO:0006606	protein import into nucleus	20	0.021272498
B2 t1 vs. B2 t2	Up	GO:0030970	retrograde protein transport, ER to cytosol	9	0.023542072
B2 t1 vs. B2 t2	Up	GO:0031398	positive regulation of protein ubiquitination	17	0.024088893
B2 t1 vs. B2 t2	Up	gga04141	Protein processing in endoplasmic reticulum	93	1.04E-07
B2 t1 vs. B2 t2	Up	gga03010	Ribosome	76	9.85E-07
B2 t1 vs. B2 t2	Up	gga04120	Ubiquitin mediated proteolysis	77	4.09E-06
B2 t1 vs. B2 t2	Up	gga03040	Spliceosome	66	1.88E-05
B2 t1 vs. B2 t2	Up	gga03018	RNA degradation	44	5.23E-05
B2 t1 vs. B2 t2	Up	gga03020	RNA polymerase	18	8.51E-05
B2 t1 vs. B2 t2	Up	gga00190	Oxidative phosphorylation	68	2.07E-04
B2 t1 vs. B2 t2	Up	gga03013	RNA transport	75	3.69E-04
B2 t1 vs. B2 t2	Up	gga03420	Nucleotide excision repair	26	6.71E-04
B2 t1 vs. B2 t2	Up	gga03060	Protein export	18	7.34E-04
B2 t1 vs. B2 t2	Up	gga00240	Pyrimidine metabolism	53	9.51E-04
B2 t1 vs. B2 t2	Up	gga00510	N-Glycan biosynthesis	30	0.002686839
B2 t1 vs. B2 t2	Up	gga04110	Cell cycle	60	0.006890719
B2 t1 vs. B2 t2	Up	gga04142	Lysosome	60	0.006890719
B2 t1 vs. B2 t2	Up	gga00071	Fatty acid degradation	21	0.021537199
B2 t1 vs. B2 t2	Up	gga03022	Basal transcription factors	23	0.021579106
B2 t1 vs. B2 t2	Up	gga03015	mRNA surveillance pathway	38	0.029025863
B2 t1 vs. B2 t2	Up	gga04144	Endocytosis	113	0.032927988
B2 t1 vs. B2 t2	Up	gga04150:	mTOR signaling pathway	28	0.039075053
B2 t1 vs. B2 t2	Up	R-GGA-5419276	Mitochondrial translation termination	40	3.56E-07
B2 t1 vs. B2 t2	Up	R-GGA-5389840	Mitochondrial translation elongation	39	6.66E-07
B2 t1 vs. B2 t2	Up	R-GGA-2467813	Separation of Sister Chromatids	57	2.33E-06
B2 t1 vs. B2 t2	Up	R-GGA-2500257:	Resolution of Sister Chromatid Cohesion	50	4.11E-06
B2 t1 vs. B2 t2	Up	R-GGA-72165	mRNA Splicing—Minor Pathway	28	4.11E-04
B2 t1 vs. B2 t2	Up	R-GGA-72086	mRNA Capping	18	6.97E-04
B2 t1 vs. B2 t2	Up	R-GGA-76042	RNA Polymerase II Transcription Initiation And Promoter Clearance	24	9.36E-05
B2 t1 vs. B2 t2	Up	R-GGA-75953	RNA Polymerase II Transcription Initiation	24	9.36E-05
B2 t1 vs. B2 t2	Up	R-GGA-73779	RNA Polymerase II Transcription Pre-Initiation And Promoter Opening	24	9.36E-05
B2 t1 vs. B2 t2	Up	R-GGA-1834949	Cytosolic sensors of pathogen-associated DNA	13	8.95E-04
B2 t1 vs. B2 t2	Up	R-GGA-166208	mTORC1-mediated signaling	10	0.007874037
B2 t1 vs. B2 t2	Up	R-GGA-5674135	MAP2K and MAPK activation	11	0.010508603
B2 t1 vs. B2 t2	Up	R-GGA-202424	Downstream TCR signaling	13	0.02759075
B2 t1 vs. B2 t2	Up	R-GGA-2871796	FCERI mediated MAPK activation	14	0.029000225
B2 t1 vs. B2 t2	Up	R-GGA-174084	Autodegradation of Cdh1 by Cdh1:APC/C	24	0.029609758
B2 t1 vs. B2 t2	Up	R-GGA-2730905	Role of LAT2/NTAL/LAB on calcium mobilization	8	0.031070119
B2 t1 vs. B2 t2	Up	R-GGA-5607764	CLEC7A (Dectin-1) signaling	10	0.041042518
B2 t2 vs. B2 t4	Down	GO:0090307	mitotic spindle assembly	5	6.17E-04
B2 t2 vs. B2 t4	Down	GO:0000070	mitotic sister chromatid segregation	4	0.001514614
B2 t2 vs. B2 t4	Down	GO:0007059	chromosome segregation	5	0.004071808
B2 t2 vs. B2 t4	Down	GO:0007094	mitotic spindle assembly checkpoint	3	0.008710879
B2 t2 vs. B2 t4	Down	GO:0046849	bone remodeling	3	0.011065539
B2 t2 vs. B2 t4	Down	GO:0006464	cellular protein modification process	3	0.013666474
B2 t2 vs. B2 t4	Down	GO:0035556	intracellular signal transduction	10	0.014298815
B2 t2 vs. B2 t4	Down	gga04110	Cell cycle	11	3.28E-05
B2 t2 vs. B2 t4	Down	R-GGA-2467813	Separation of Sister Chromatids	11	2.41E-05
B2 t2 vs. B2 t4	Down	R-GGA-5663220	RHO GTPases Activate Formins	10	3.80E-05
B2 t2 vs. B2 t4	Down	R-GGA-2500257	Resolution of Sister Chromatid Cohesion	10	4.26E-05
B2 t2 vs. B2 t4	Down	R-GGA-5620912	Anchoring of the basal body to the plasma membrane	6	0.016443642
B2 t2 vs. B2 t4	Down	R-GGA-5693568	Resolution of D-loop Structures through Holliday Junction Intermediates	4	0.016488095
B2 t2 vs. B2 t4	Down	R-GGA-5620922	BBSome-mediated cargo-targeting to cilium	3	0.022369863
B2 t2 vs. B2 t4	Down	R-GGA-69205	G1/S-Specific Transcription	3	0.026920963
B2 t2 vs. B2 t4	Down	R-GGA-113510	E2F mediated regulation of DNA replication	3	0.026920963
B2 t2 vs. B2 t4	Down	R-GGA-176187	Activation of ATR in response to replication stress	4	0.027999251
B2 t2 vs. B2 t4	Down	R-GGA-606279	Deposition of new CENPA-containing nucleosomes at the centromere	4	0.03650445
B2 t2 vs. B2 t4	Down	R-GGA-2565942	Regulation of PLK1 Activity at G2/M Transition	5	0.038794821
B2 t2 vs. B2 t4	Up	GO:0006955	immune response	12	1.35E-07
B2 t2 vs. B2 t4	Up	GO:0006954	inflammatory response	11	4.10E-06
B2 t2 vs. B2 t4	Up	GO:0032496	response to lipopolysaccharide	8	4.67E-06
B2 t2 vs. B2 t4	Up	GO:0042832	defense response to protozoan	3	0.004389284
B2 t2 vs. B2 t4	Up	GO:0051607	defense response to virus	5	0.004729744
B2 t2 vs. B2 t4	Up	GO:0097190	apoptotic signaling pathway	4	0.004879708
B2 t2 vs. B2 t4	Up	GO:0042981	regulation of apoptotic process	6	0.007547916
B2 t2 vs. B2 t4	Up	GO:0032735	positive regulation of interleukin-12 production	3	0.008406525
B2 t2 vs. B2 t4	Up	GO:0045071	negative regulation of viral genome replication	3	0.008406525
B2 t2 vs. B2 t4	Up	GO:0042742	defense response to bacterium	4	0.008606681
B2 t2 vs. B2 t4	Up	GO:0043410	positive regulation of MAPK cascade	4	0.009352126
B2 t2 vs. B2 t4	Up	GO:0042127	regulation of cell proliferation	6	0.011369564
B2 t2 vs. B2 t4	Up	GO:0050717	positive regulation of interleukin-1 alpha secretion	2	0.025630045
B2 t2 vs. B2 t4	Up	GO:0048873	homeostasis of number of cells within a tissue	3	0.026931634
B2 t2 vs. B2 t4	Up	gga04060	Cytokine-cytokine receptor interaction	13	5.07E-07
B2 t2 vs. B2 t4	Up	gga05164	Influenza A	7	0.004668633
B2 t4 vs. B2 t8	Down	GO:0042787	protein ubiquitination involved in ubiquitin-dependent protein catabolic process	63	1.69E-07
B2 t4 vs. B2 t8	Down	GO:0006511	ubiquitin-dependent protein catabolic process	55	9.75E-07
B2 t4 vs. B2 t8	Down	GO:0030433	ER-associated ubiquitin-dependent protein catabolic process	30	3.12E-06
B2 t4 vs. B2 t8	Down	GO:0006886	intracellular protein transport	81	5.55E-06
B2 t4 vs. B2 t8	Down	GO:0045454	cell redox homeostasis	35	4.20E-05
B2 t4 vs. B2 t8	Down	GO:0006888	ER to Golgi vesicle-mediated transport	31	5.01E-05
B2 t4 vs. B2 t8	Down	GO:0007030	Golgi organization	36	8.73E-05
B2 t4 vs. B2 t8	Down	GO:0050821	protein stabilization	43	1.27E-04
B2 t4 vs. B2 t8	Down	GO:0000398	mRNA splicing, via spliceosome	35	2.60E-04
B2 t4 vs. B2 t8	Down	GO:0006457	protein folding	48	3.44E-04
B2 t4 vs. B2 t8	Down	GO:0006360	transcription from RNA polymerase I promoter	12	3.71E-04
B2 t4 vs. B2 t8	Down	GO:0000209	protein polyubiquitination	37	4.73E-04
B2 t4 vs. B2 t8	Down	GO:0015031	protein transport	43	5.18E-04
B2 t4 vs. B2 t8	Down	GO:0008333	endosome to lysosome transport	19	6.62E-04
B2 t4 vs. B2 t8	Down	GO:0043161	proteasome-mediated ubiquitin-dependent protein catabolic process	48	7.64E-04
B2 t4 vs. B2 t8	Down	GO:0019827	stem cell population maintenance	21	0.010108895
B2 t4 vs. B2 t8	Down	GO:0007049	cell cycle	26	0.010146854
B2 t4 vs. B2 t8	Down	GO:0031929	TOR signaling	10	0.017015043
B2 t4 vs. B2 t8	Down	gga04141	Protein processing in endoplasmic reticulum	98	8.59E-09
B2 t4 vs. B2 t8	Down	gga03010	Ribosome	78	9.30E-07
B2 t4 vs. B2 t8	Down	gga04142	Lysosome	73	1.78E-06
B2 t4 vs. B2 t8	Down	gga03040	Spliceosome	69	6.04E-06
B2 t4 vs. B2 t8	Down	gga00190	Oxidative phosphorylation	72	4.26E-05
B2 t4 vs. B2 t8	Down	gga03013	RNA transport	77	4.14E-04
B2 t4 vs. B2 t8	Down	gga04120	Ubiquitin mediated proteolysis	72	7.75E-04
B2 t4 vs. B2 t8	Down	gga03020	RNA polymerase	17	9.00E-04
B2 t4 vs. B2 t8	Down	gga03050	Proteasome	26	0.001325244
B2 t4 vs. B2 t8	Down	gga00510	N-Glycan biosynthesis	31	0.002147473
B2 t4 vs. B2 t8	Down	gga03018	RNA degradation	41	0.002187297
B2 t4 vs. B2 t8	Down	gga03015	mRNA surveillance pathway	43	0.002311437
B2 t4 vs. B2 t8	Down	gga00071	Fatty acid degradation	23	0.005623955
B2 t4 vs. B2 t8	Down	gga04150	mTOR signaling pathway	30	0.017796114
B2 t4 vs. B2 t8	Down	gga04144	Endocytosis	118	0.023369828
B2 t4 vs. B2 t8	Down	gga00562	Inositol phosphate metabolism	39	0.023965717
B2 t4 vs. B2 t8	Down	gga00240	Pyrimidine metabolism	49	0.026875801
B2 t4 vs. B2 t8	Down	gga01100	Metabolic pathways	489	0.030757983
B2 t4 vs. B2 t8	Down	gga03022	Basal transcription factors	23	0.03456006
B2 t4 vs. B2 t8	Down	gga04621	NOD-like receptor signaling pathway	25	0.03499728
B2 t4 vs. B2 t8	Down	gga04068	FoxO signaling pathway	63	0.03789817
B2 t4 vs. B2 t8	Down	R-GGA-73762	RNA Polymerase I Transcription Initiation	29	4.07E-08
B2 t4 vs. B2 t8	Down	R-GGA-72163	mRNA Splicing—Major Pathway	68	6.37E-08
B2 t4 vs. B2 t8	Down	R-GGA-5419276	Mitochondrial translation termination	41	2.05E-07
B2 t4 vs. B2 t8	Down	R-GGA-73772	RNA Polymerase I Promoter Escape	22	1.56E-06
B2 t4 vs. B2 t8	Down	R-GGA-5389840	Mitochondrial translation elongation	39	1.73E-06
B2 t4 vs. B2 t8	Down	R-GGA-674695	RNA Polymerase II Pre-transcription Events	38	3.11E-06
B2 t4 vs. B2 t8	Down	R-GGA-1799339	SRP-dependent cotranslational protein targeting to membrane	45	6.11E-06
B2 t4 vs. B2 t8	Down	R-GGA-73863	RNA Polymerase I Transcription Termination	21	1.53E-05
B2 t4 vs. B2 t8	Down	R-GGA-6781823	Formation of TC-NER Pre-Incision Complex	32	1.93E-05
B2 t4 vs. B2 t8	Down	R-GGA-975957	Nonsense Mediated Decay (NMD) enhanced by the Exon Junction Complex (EJC)	50	2.13E-05
B2 t4 vs. B2 t8	Down	R-GGA-73779	RNA Polymerase II Transcription Pre-Initiation And Promoter Opening	25	3.47E-05
B2 t4 vs. B2 t8	Down	R-GGA-75953	RNA Polymerase II Transcription Initiation	25	3.47E-05
B2 t4 vs. B2 t8	Down	R-GGA-76042	RNA Polymerase II Transcription Initiation And Promoter Clearance	25	3.47E-05
B2 t4 vs. B2 t8	Down	R-GGA-72706	GTP hydrolysis and joining of the 60S ribosomal subunit	41	4.75E-05
B2 t4 vs. B2 t8	Down	R-GGA-975956	Nonsense Mediated Decay (NMD) independent of the Exon Junction Complex (EJC)	43	5.97E-05
B2 t4 vs. B2 t8	Down	R-GGA-75955	RNA Polymerase II Transcription Elongation	27	6.68E-05
B2 t4 vs. B2 t8	Down	R-GGA-5696395	Formation of Incision Complex in GG-NER	25	9.14E-05
B2 t4 vs. B2 t8	Down	R-GGA-6782210	Gap-filling DNA repair synthesis and ligation in TC-NER	32	9.24E-05
B2 t4 vs. B2 t8	Down	R-GGA-6782135	Dual incision in TC-NER	33	1.10E-04
B2 t4 vs. B2 t8	Down	R-GGA-72086	mRNA Capping	19	2.11E-04
B2 t4 vs. B2 t8	Down	R-GGA-72165	mRNA Splicing—Minor Pathway	28	7.54E-04
B2 t4 vs. B2 t8	Down	R-GGA-204005	COPII (Coat Protein 2) Mediated Vesicle Transport	30	0.006870202
B2 t4 vs. B2 t8	Down	R-GGA-1834949	Cytosolic sensors of pathogen-associated DNA	12	0.007282044
B2 t4 vs. B2 t8	Down	R-GGA-983168	Antigen processing: Ubiquitination & Proteasome degradation	34	0.007438297
B2 t4 vs. B2 t8	Down	R-GGA-202424	Downstream TCR signaling	14	0.011275517
B2 t4 vs. B2 t8	Down	R-GGA-917729	Endosomal Sorting Complex Required For Transport (ESCRT)	14	0.011275517
B2 t4 vs. B2 t8	Down	R-GGA-2730905	Role of LAT2/NTAL/LAB on calcium mobilization	8	0.037401415
B2 t4 vs. B2 t8	Down	R-GGA-2871796	FCERI mediated MAPK activation	14	0.038198199
B2 t4 vs. B2 t8	Down	R-GGA-109688	Cleavage of Growing Transcript in the Termination Region	23	0.043691582
B2 t4 vs. B2 t8	Down	R-GGA-166208	mTORC1-mediated signaling	9	0.044990961
B2 t4 vs. B2 t8	Down	R-GGA-1445148	Translocation of GLUT4 to the plasma membrane	9	0.044990961
B2 t8 vs. B2 t16	Down	GO:0010634	positive regulation of epithelial cell migration	4	7.45E-05
B2 t8 vs. B2 t16	Down	GO:0045747	positive regulation of Notch signaling pathway	4	2.45E-04
B2 t8 vs. B2 t16	Down	GO:0006955	immune response	6	3.59E-04
B2 t8 vs. B2 t16	Down	GO:0006954	inflammatory response	6	6.50E-04
B2 t8 vs. B2 t16	Down	GO:0032735	positive regulation of interleukin-12 production	3	0.001678191
B2 t8 vs. B2 t16	Down	GO:0040008	regulation of growth	3	0.002746603
B2 t8 vs. B2 t16	Down	GO:0051607	defense response to virus	4	0.00329606
B2 t8 vs. B2 t16	Down	GO:2000379	positive regulation of reactive oxygen species metabolic process	3	0.004551512
B2 t8 vs. B2 t16	Down	GO:0018107	peptidyl-threonine phosphorylation	3	0.008034447
B2 t8 vs. B2 t16	Down	GO:0090002	establishment of protein localization to plasma membrane	3	0.010846074
B2 t8 vs. B2 t16	Down	GO:0018401	peptidyl-proline hydroxylation to 4-hydroxy-L-proline	2	0.016916932
B2 t8 vs. B2 t16	Down	GO:0007050	cell cycle arrest	3	0.017563794
B2 t8 vs. B2 t16	Down	GO:2000107	negative regulation of leukocyte apoptotic process	2	0.022493312
B2 t8 vs. B2 t16	Down	GO:0007179	transforming growth factor beta receptor signaling pathway	3	0.026723558
B2 t8 vs. B2 t16	Down	GO:0070102	interleukin-6-mediated signaling pathway	2	0.028038678
B2 t8 vs. B2 t16	Down	GO:2000505	regulation of energy homeostasis	2	0.033553199
B2 t8 vs. B2 t16	Down	GO:0009612	response to mechanical stimulus	2	0.033553199
B2 t8 vs. B2 t16	Down	GO:0032496	response to lipopolysaccharide	3	0.036148438
B2 t8 vs. B2 t16	Down	gga04060	Cytokine-cytokine receptor interaction	9	2.22E-05
B2 t8 vs. B2 t16	Down	gga04630	Jak-STAT signaling pathway	5	0.010473786
B2 t16 vs. B2 t24	Down	GO:0002540	leukotriene production involved in inflammatory response	2	0.002222592
B2 t16 vs. B2 t24	Down	GO:0019370	leukotriene biosynthetic process	2	0.007759634
B2 t16 vs. B2 t24	Up	GO:0060612	adipose tissue development	5	2.03E-04
B2 t16 vs. B2 t24	Up	GO:0070373	negative regulation of ERK1 and ERK2 cascade	5	0.003254965
B2 t16 vs. B2 t24	Up	GO:0007264	small GTPase mediated signal transduction	10	0.00727077
B2 t16 vs. B2 t24	Up	GO:0031589	cell-substrate adhesion	3	0.016133553
B2 t16 vs. B2 t24	Up	GO:0033138	positive regulation of peptidyl-serine phosphorylation	5	0.016364796
B2 t16 vs. B2 t24	Up	GO:0016477	cell migration	7	0.01765893
B2 t16 vs. B2 t24	Up	GO:0043277	apoptotic cell clearance	3	0.019873166
B2 t16 vs. B2 t24	Up	GO:0032720	negative regulation of tumor necrosis factor production	3	0.043108056

Enrichment analysis was performed with gene expression data associated with the B2 haplotype. Enrichment was calculated for genes exhibiting statistically significant differences in expression across the successive time points (S1 Table). Three distinct annotation databases were used for enrichment analysis: Gene Ontology—Biological Process, KEGG Pathways, and Reactome Pathways. Complete enrichment annotation available in S2, S3 and S4 Tables.

Between the -6 day and -3 day time points, a large number of genes exhibit reduced expression. These genes are enriched for biological processes such as transcription, mRNA splicing, tRNA processing and negative regulation NFκB mediated gene expression. Similarly, KEGG pathways enriched include RNA polymerase, RNA degradation, Metabolic pathways and Basal transcription factors. The reactome enriched pathways mirror these results with annotations of RNA polymerase II initiation, mRNA capping, and some immune functions including antigen presentation and cytosolic sensors of pathogen-associated DNA.

The transition from day -3 to t0 (just prior to IFNγ stimulation) correlates with down regulation of genes associated with chromosome segregation, mitotic nuclear division and DNA repair, cell cycle pathways, acetylation and some immunological functions of interleukin 3 and 5 signaling and interleukin receptor signaling. Genes exhibiting increases in expression during this interval were enriched in processes and pathways related to interleukin 4, biosynthesis of amino acids, and metabolic pathways. Within an hour of IFNγ stimulation genes exhibiting increased expression were associated with inflammatory and defense responses, toll-like receptor signaling pathways, cell chemotaxis, MyD88 signaling, Jak-STAT signaling, cell adhesion, FoxO signaling, and raf activation.

Genes exhibiting an increase in expression within two hours of IFNγ stimulation are enriched for biological processes of intracellular protein transport, ER-to-Golgi vesical mediated transport, endosome to lysosomal transport, chromatin remodeling, histone H3 acetylation, regulation of vesicle fusion and protein import into the nucleus. Among the KEGG pathways that exhibit enrichment for these genes are RNA transport, protein export, lysosome, mRNA surveillance pathway, endocytosis and mTOR signaling. Similarly, the enriched reactome pathways mirror these processes and include cytosolic sensors of pathogen-associated DNA, RNA polymerase II initiation and promoter clearance, mTORC1-mediated signaling, MAP2K and MAPK activation, downstream TCR signaling, FCε Receptor 1 mediated MAPK activation, and Clec7A signaling.

Genes exhibiting decreased expression between 2 hours and 4 hours following IFNγ stimulation correspond to reduced expression of cell-cycle pathways and mediators, as well as genes implicated in G1/S transcription, DNA replication, and separation of sister chromatids. Biological processes identified within these genes include mitotic spindle assembly, chromosome segregation, and mitotic spindle checkpoint assembly. Conversely, genes exhibiting increased expression during this same time are enriched for biological processes of inflammatory response, defense response to virus, positive regulation of interleukin 12 production, negative replication of viral genome replication, bacterial defense processes and positive regulation of IL1α secretion. KEGG pathways associated with these genes include cytokine-cytokine receptor interaction and genes implicated in influenza A signaling. Reactome processes identified included stem cell population maintenance and TOR signaling.

Over the remaining time points, from 4 hours to 8 hours, from 8 hours to 16 hours and from 16 hours to 24 hours the B2 cells exhibit a systemic down regulation of the genes that were initially activated during the IFNγ stimulation. Overall, the gene enrichment analysis of the RNA sequence data provides a cellular-level picture of the specific biological processes that occur over time following activation of monocyte-derived macrophages.

## Discussion

Previous work in our laboratories investigated the association between chicken haplotype and disease resistance, specifically the enhanced resistance of B2 haplotypes to avian coronavirus IBV [10] and the influence of innate immunity leading to decreased clinical signs of illness. We showed that macrophages play an important role in this enhanced immunity, demonstrating much better activation in response to stimulation [13]. To analyze the gene expression involved in this process leading to increased macrophage nitric oxide release in B2 haplotypes, we stimulated macrophages from B2 and B19 chicks for RNA sequencing. In addition, we had observed different cell morphology when isolated monocytes from B2 and B19 haplotypes were differentiating into macrophages and therefore time points before stimulation, during differentiation of the macrophages, were included in this study.

The rationale for investigating the gene expression differences between the B2 and B19 haplotype birds was to address underlying questions that were raised at the end of our previous studies: 1. *Why do the IFNγ stimulated B19 derived macrophages exhibit decreased nitric oxide production compared to the IFNγ stimulated B2 derived macrophages*? 2. *How do the two lineages of macrophages differ at the gene expression level*? 3. *What specific patterns of gene expression correlate with divergent macrophage differentiation*, *activation and function*? 4. *What is the underlying cause of the divergent gene expression patterns observed between B2 and B19 macrophages*?

The data collection, analysis and interpretation of results described herein provide plausible answers to these questions based on bioinformatics and functional genomics approaches. Although these answers are more realistically new hypotheses for further investigation, they do represent significant advances in the understanding of B2 and B19 monocyte differentiation into macrophages and the resulting divergent patterns of B2 and B19 macrophage activation and function.

Ultimately, the findings and interpretations we report must be functionally and experimentally validated. Even so, the use of computational methods to answer these questions represents a valuable first step in deciphering the cellular phenotypes underlying MHC haplotype variation in macrophage cells.

Our results demonstrate that there are large numbers of genes differentially expressed in the two haplotypes, both during differentiation of peripheral monocytes into mature macrophages, as well as after stimulation of differentiated macrophages with interferon.

The answer to the question of why the IFNγ stimulated B2 haplotype cells produce more nitric oxide than the IFNγ stimulated B19 cells lies in the timing of macrophage differentiation and the phenotypic variation that is set up early prior to IFNγ stimulation, such as divergent expression of genes involved in differentiation and immune competence. At day t-6, after plating of monocytes without interferon stimulation, several genes relating to inflammation, interferon responses and differentiation are upregulated in the B2 haplotype. This is to be expected as adherence of the monocytes is actually an activation signal, but it is notable that this signal is not resulting in the same gene expression pattern in the B19 haplotype. This pattern can be observed for genes such as IL1β, PTSG2, IL6, which are mainly associated with the inflammatory M1 phenotype. On the other hand, Adenosine receptor A2B is also showing increased gene expression at this time in B2 cells, and this receptor plays an important role in differentiation, as well as in the inflammatory response. Expressions of these genes are consequently initially increased at the time of adherence, and then again after stimulation with interferon, in the B2 haplotype. Some, but not all of these genes are expressed after stimulation with interferon in the B19 haplotype, but not to the same extent as the B2 macrophages, which appears to relate to the initial lack of expression at t-6 days, the beginning of differentiation.

Another interesting observation was the differential expression of genes at day t-6 versus day t-3 in the two haplotypes, as a large number of genes is highly expressed in both haplotypes at day t-6, but then is completely shut down in B2 haplotypes while showing delayed expression until day t-3 in the B19 birds. This seems to indicate a lack of appropriate regulation in the B19 birds, consequently leading to less coherent initiation of gene expression when stimulated. Some of the genes showing this pattern are macrophage differentiation associated GATA2 and FADD, as well as macrophage podosome markers VCL and GSN. Taken together, these results appear to suggest that the regulation of B2 differentiation from monocyte to macrophage is very tightly regulated with many genes increasing in expression, quickly followed by shutting down this increased gene expression. In contrast, the regulation of B19 gene expression is not well regulated, appearing to “linger” with either delayed or extended gene expression. Consequently, we observed differences in expression of genes after stimulation with interferon. Specifically, genes that were strongly expressed at day t-6 and not expressed (or only weakly expressed) at day t-3 in the B2 birds, were robustly increased at 2 and 4 hours of stimulation. In contrast, the same genes showed weak and delayed expression in B19 birds after stimulation, emphasizing the importance of the regulation of gene expression during differentiation. This relates very well to the differences we previously reported in morphology of B2 and B19 macrophages during differentiation and after stimulation.

Our results provide insight into the complexity associated with macrophage differentiation, activation and function. Thousands of genes are up-regulated and then down-regulated in a 24-hour period following IFNγ stimulation. The coordinated activity of multiple regulatory and gene expression control mechanisms is required to effectively achieve the dramatic changes in internal cellular programing that occur. Although the B2 and B19 birds’ haplotypes differ within the MHC locus, the functional consequences of this genetic difference extend well beyond the genes encoded within the B-locus, including macrophage differentiation, M1 and M2 macrophage markers, lysosomal factors involved in phagocytosis, podosome development, invadosome capabilities, chemotaxis potential and matrix degradation ability. Additionally, during the process of differentiation and activation, thousands of genes associated with basic cellular biology undergo rapid changes in expression in coordination with the expression of factors associated with cell renewal and proliferation such as cell cycle regulators, mitotic spindle components, factors involved in chromatin remodeling, molecules required for chromosome segregation and nuclear division.

Taken together, our data and interpretations provide a framework of possible mechanisms of B2 and B19 macrophage biology in differentiation and activation. As such, our findings offer a number of hypotheses about macrophage cell biology that can be used for subsequent studies aimed at validating our findings. Although we performed RT-PCR on a set of differentially expressed genes between the B2 and B19 macrophages, it is not feasible, or possible, to systematically verify, via RT-PCR, each and every transcript, observed at each time point, in the experiment. Even so, our PCR validation provides independent evidence that the pattern of gene expression we observed in the RNA sequence data, was consistent and reproducible which is also in line with our previous research detailing differences in macrophage activation and function [13]

## Conclusions

We have tried to elucidate possible mechanisms involved in enhanced disease resistance and macrophage functions displayed by B2 haplotype chickens compared to B19. This study highlights the complex gene expression patterns involved in macrophage differentiation and activation.

One of the main conclusions from the large number of differences seen in the gene expression of the two haplotypes is the fact that there are not just a few genes or genetic markers that can be readily identified as being the ultimate cause of enhanced macrophage function in B2 chickens. Rather, it appears that events during differentiation of monocytes into macrophages have a significant impact on the subsequent ability for stimulation of immune genes after IFNγ treatment. The differences in gene expression correlate with the previously observed differences in morphology of the two haplotypes, with B2 macrophages having a more typical macrophage appearance [13].

Considering the global temporal dysregulation of many genes in B19 haplotypes compared to the more resistant B2 chicks, it seems likely that a variety of genomic regulatory mechanisms (such as transcription factors, miRNAs, snoRNAs, ubiquitin mediated proteasomal degradation, and epigenetic regulation) might play a major role in this process which will be further detailed in a future publication. It will be of great interest to further elucidate these mechanisms and their connection to enhanced immunity. Ultimately, our detailed model of macrophage differentiation, activation and function following IFNγ stimulation provides a high resolution molecular map of cellular biology which can be leveraged by other investigators to further explore the role of these genes in immunology.

## Supporting information

S1 Table Significant differences in gene expression with P-Values. Pairwise gene expression differences between samples (B2 and B19 haplotype) and timepoints (-6 days, -3 days, 0 days, 1 hr, 2 hrs, 4 hrs, 8 hrs, 16 hrs, 24 hrs) are provided with p-values in excel format. The analysis described within this manuscript focused on differences between [1] matched time points between B2 and B19 haplotype chickens (such as B2 1 hr versus B19 1 hr) as well as [2] progressive timepoints within the same haplotype group (such as B2 1hr versus B2 2hr and B19 4 hr versus B19 8 hr). Subsequently the data contained in this supplemental file also focuses predominantly on those comparisons. This file contains a total of 163,043 rows including the header line containing field names (***geneName***—identifier for each gene either as gene symbol or ensemble geneId; ***locus***—chromosome number and the start-end base pair location of the gene; ***sample1*** and ***sample2***—the “paired” samples compared for significant gene expression, note ‘AB’ corresponds to B2 haplotype and ‘EC’ corresponds to B19 haplotype; ***testStatus***—indication that the analysis method performed by cuffdiff program within the cufflinks package was ‘OK’; ***fpkm1*** and ***fpkm2***—the fpkm values for sample 1 and sample 2 respectively; ***log2fpkm***—the log of the ratio of fpkm1 and fpkm2; *testStat*—the test statistic generated during the statistical analysis; ***pValue***—the p-value corresponding to the difference in expression between sample1 and sample2; ***qValue***—a multiple testing corrected p-value; ***signif***—‘yes’ indicates that the pairwise difference in expression is in fact statistically significant).
(XLSX)

S2 Table Significant gene ontology biological process enrichment analysis results. Enriched gene ontology biological process terms identified within up and down regulated genes within the B2 haplotype chicken samples across progressive time points (-6 days, -3 days, 0 days, 1 hr, 2 hrs, 4 hrs, 8 hrs, 16 hrs, 24 hrs) are provided in excel file format. Since the B2 chickens exhibited the most robust macrophage phenotype these samples were used for the analysis as a means of characterizing the biological processes that were associated with the altered gene expression across the experimental time points. Note ‘AB’ indicates B2 haplotype. The file contains a total of 362 rows including the header line containing field names (***Sample Comparison***—indicates the specific pair of time points for which gene expression changes were identified; ***Gene Set***—indicates the specific set of differentially expressed genes, ‘Down’ or ‘Up’; ***Category***—indicates the specific subset of terms that were used for the analysis; ***Term***—provides the gene ontology identifier for the identified gene ontology term/annotation; ***Description***—the specific enriched gene ontology biological process term; ***Gene Count***—the number of genes within the differentially expressed genes that are mapped to the particular enriched gene ontology term; ***%***—the corresponding percent associated with the specific number of genes; ***P-Value***—the p-value associated with the gene ontology term enrichment).
(XLSX)

S3 Table Significant KEGG pathway enrichment analysis results. Since the B2 chickens exhibited the most robust macrophage phenotype these samples were used for the analysis as a means of characterizing the KEGG pathways that were associated with the altered gene expression across the experimental time points. Note ‘AB’ indicates B2 haplotype. The file contains a total of 110 rows including the header line containing field names (***Sample Comparison***—indicates the specific pair of time points for which gene expression changes were identified; ***Gene Set***—indicates the specific set of differentially expressed genes, ‘Down’ or ‘Up’; ***Category***—indicates the specific subset of terms that were used for the analysis; ***Term***—provides the KEGG pathway identifier for the identified pathway term/annotation; ***Description***—the specific enriched KEGG pathway term; ***Gene Count***—the number of genes within the differentially expressed genes that are mapped to the particular enriched KEGG pathway term; ***%***—the corresponding percent associated with the specific number of genes; ***P-Value***—the p-value associated with the KEGG pathway term enrichment).
(XLSX)

S4 Table Significant reactome pathway enrichment analysis results. Since the B2 chickens exhibited the most robust macrophage phenotype these samples were used for the analysis as a means of characterizing the Reactome pathways that were associated with the altered gene expression across the experimental time points. Note ‘AB’ indicates B2 haplotype. The file contains a total of 110 rows including the header line containing field names (***Sample Comparison***—indicates the specific pair of time points for which gene expression changes were identified; ***Gene Set***—indicates the specific set of differentially expressed genes, ‘Down’ or ‘Up’; ***Category***—indicates the specific subset of terms that were used for the analysis; ***Term***—provides the Reactome pathway identifier for the identified pathway term/annotation; ***Description***—the specific enriched Reactome pathway term; ***Gene Count***—the number of genes within the differentially expressed genes that are mapped to the particular enriched Reactome pathway term; ***%***—the corresponding percent associated with the specific number of genes; ***P-Value***—the p-value associated with the Reactome pathway term enrichment).
(XLSX)

## References

[1] Malek M.; Lamont S.J. Association of INOS, TRAIL, TGF-beta2, TGF-beta3, and IgL genes with response to Salmonella enteritidis in poultry. Genetics, selection, evolution: GSE. 2003;35 Suppl 1:S99–111. 10.1186/1297-9686-35-S1-S99 PMC3231766 12927083

[2] Shi K.Q.; Cai X.H.; Xiao D.D.; Wu S.J.; Peng M.M.; Lin X.F.; Liu W.Y.; Fan Y.C.; Chen Y.P.; Zheng M.H. Tumour necrosis factor-alpha-857T allele reduces the risk of hepatitis B virus infection in an Asian population. Journal of viral hepatitis. 2012 2;19(2):e66–72. doi: 10.1111/j.1365-2893.2011.01540.x 22239528 10.1111/j.1365-2893.2011.01540.x

[3] Ferro P.J.; Swaggerty C.L.; Kaiser P.; Pevzner I.Y.; Kogut M.H. Heterophils isolated from chickens resistant to extra-intestinal Salmonella enteritidis infection express higher levels of pro-inflammatory cytokine mRNA following infection than heterophils from susceptible chickens. Epidemiol Infect. 2004 12;132(6):1029–1037. 15635959 10.1017/s0950268804002687 PMC2870193

[4] Swaggerty C.L.; Pevzner I.Y.; Kaiser P.; Kogut M.H. Profiling pro-inflammatory cytokine and chemokine mRNA expression levels as a novel method for selection of increased innate immune responsiveness. Vet Immunol Immunopathol. 2008 11 15;126(1–2):35–42. doi: 10.1016/j.vetimm.2008.06.005 18656269 10.1016/j.vetimm.2008.06.005

[5] Yoo B.H.; Sheldon B.L. Association of the major histocompatibility complex with avian leukosis virus infection in chickens. Br Poult Sci. 1992 7;33(3):613–620. doi: 10.1080/00071669208417500 1322760 10.1080/00071669208417500

[6] Heinzelmann E.W.; Clark K.K.; Collins W.M.; Briles W.E. Host age and major histocompatibility genotype influence on Rous sarcoma regression in chickens. Poult Sci. 1981 10;60(10):2171–2175. 6276876 10.3382/ps.0602171

[7] Schat K.A.; Taylor R.L. Jr.; Briles W.E. Resistance to Marek's disease in chickens with recombinant haplotypes to the major histocompatibility (B) complex. Poult Sci. 1994 4;73(4):502–508. 8202429 10.3382/ps.0730502

[8] Kaufman J.; Wallny H.J. Chicken MHC molecules, disease resistance and the evolutionary origin of birds. Curr Top Microbiol Immunol. 1996;212:129–141. 8934816 10.1007/978-3-642-80057-3_12

[9] Hunt H.D.; Jadhao S.; Swayne D.E. Major histocompatibility complex and background genes in chickens influence susceptibility to high pathogenicity avian influenza virus. Avian Dis. 2010 3;54(1 Suppl):572–575. doi: 10.1637/8888-042409-ResNote.1 20521696 10.1637/8888-042409-ResNote.1

[10] Banat G.R.; Tkalcic S.; Dzielawa J.A.; Jackwood M.W.; Saggese M.D.; Yates L.; Kopulos R.; Briles W.E.; Collisson E.W. Association of the chicken MHC B haplotypes with resistance to avian coronavirus. Dev Comp Immunol. 2013 4;39(4):430–437. doi: 10.1016/j.dci.2012.10.006 23178407 10.1016/j.dci.2012.10.006 PMC7103219

[11] Kim D.K.; Lillehoj H.S.; Hong Y.H.; Park D.W.; Lamont S.J.; Han J.Y.; Lillehoj E.P. Immune-related gene expression in two B-complex disparate genetically inbred Fayoumi chicken lines following Eimeria maxima infection. Poult Sci. 2008 3;87(3):433–443. doi: 10.3382/ps.2007-00383 18281568 10.3382/ps.2007-00383

[12] Lamont S.J. The chicken major histocompatibility complex in disease resistance and poultry breeding. J Dairy Sci. 1989 5;72(5):1328–1333. doi: 10.3168/jds.S0022-0302(89)79240-7 2568373 10.3168/jds.S0022-0302(89)79240-7

[13] Dawes M.E.; Griggs L.M.; Collisson E.W.; Briles W.E.; Drechsler Y. Dramatic differences in the response of macrophages from B2 and B19 MHC-defined haplotypes to interferon gamma and polyinosinic:polycytidylic acid stimulation. Poult Sci. 2014 4;93(4):830–838. doi: 10.3382/ps.2013-03511 24706959 10.3382/ps.2013-03511 PMC7107093

[14] Collisson E, Griggs L, Drechsler Y. Macrophages from disease resistant B2 haplotype chickens activate T lymphocytes more effectively than macrophages from disease susceptible B19 birds. Dev Comp Immunol. 2017 2;67:249–256. doi: 10.1016/j.dci.2016.09.013 27746172 10.1016/j.dci.2016.09.013 PMC7102680

[15] Qureshi T.; Templeton J.W.; Adams L.G. Intracellular survival of Brucella abortus, Mycobacterium bovis BCG, Salmonella dublin, and Salmonella typhimurium in macrophages from cattle genetically resistant to Brucella abortus. Vet Immunol Immunopathol. 1996 3;50(1–2):55–65. 9157686 10.1016/0165-2427(95)05492-8

[16] Bellamy R. Susceptibility to mycobacterial infections: the importance of host genetics. Genes and immunity. 2003 1;4(1):4–11. doi: 10.1038/sj.gene.6363915 12595896 10.1038/sj.gene.6363915

[17] Van Erp K, Dach K., Koch I., Heesemann J., Hoffmann R. (2006) Role of strain differences on host resistance and the transcriptional response of macrophages to infection with Yersinia enterocolitica. Physiol. Genomics 25, 75–84. doi: 10.1152/physiolgenomics.00188.2005 16352694 10.1152/physiolgenomics.00188.2005

[18] Castillo-Velazquez U.; Gomez-Flores R.; Tamez-Guerra R.; Tamez-Guerra P.; Rodriguez-Padilla C. Differential responses of macrophages from bovines naturally resistant or susceptible to Mycobacterium bovis after classical and alternative activation. Vet Immunol Immunopathol. 2013 7 15;154(1–2):8–16. doi: 10.1016/j.vetimm.2013.04.010 23707003 10.1016/j.vetimm.2013.04.010

[19] Raza S, Barnett MW, Barnett-Itzhaki Z, Amit I, Hume DA, Freeman TC. Analysis of the transcriptional networks underpinning the activation of murine macrophages by inflammatory mediators. J Leukoc Biol. 2014 8;96(2):167–83. doi: 10.1189/jlb.6HI0313-169R 24721704 10.1189/jlb.6HI0313-169R PMC4378362

[20] Turchetti AP, da Costa LF, Romão Ede L, Fujiwara RT, da Paixão TA, Santos RL. Transcription of innate immunity genes and cytokine secretion by canine macrophages resistant or susceptible to intracellular survival of Leishmania infantum. Vet Immunol Immunopathol. 2015 1 15;163(1–2):67–76. doi: 10.1016/j.vetimm.2014.11.010 25466388 10.1016/j.vetimm.2014.11.010

[21] Medzhitov R.; Janeway C.A. Jr. Innate immunity: the virtues of a nonclonal system of recognition. Cell. 1997a 10 31;91(3):295–298. 9363937 10.1016/s0092-8674(00)80412-2

[22] Medzhitov R.; Janeway C.A. Jr. Innate immunity: impact on the adaptive immune response. Curr Opin Immunol. 1997b 2;9(1):4–9. 9039775 10.1016/s0952-7915(97)80152-5

[23] Medzhitov R.; Janeway C. Jr. Innate immune recognition: mechanisms and pathways. Immunol Rev. 2000 2;173:89–97. 10719670 10.1034/j.1600-065x.2000.917309.x

[24] Sweet M.J.; Hume D.A. CSF-1 as a regulator of macrophage activation and immune responses. Archivum immunologiae et therapiae experimentalis. 2003;51(3):169–177. 12894871

[25] Schroder K.; Spille M.; Pilz A.; Lattin J.; Bode K.A.; Irvine K.M.; Burrows A.D.; Ravasi T.; Weighardt H.; Stacey K.J.; Decker T.; Hume D.A.; Dalpke A.H.; Sweet M.J. Differential effects of CpG DNA on IFN-beta induction and STAT1 activation in murine macrophages versus dendritic cells: alternatively activated STAT1 negatively regulates TLR signaling in macrophages. J Immunol. 2007b 9 15;179(6):3495–3503. 17785783 10.4049/jimmunol.179.6.3495

[26] Biswas S. K., Chittezhath M, Shalova I. N., Lim J. Y. (2012) Macrophage polarization and plasticity in health and disease. Immunol. Res. 53, 11–24 doi: 10.1007/s12026-012-8291-9 22418728 10.1007/s12026-012-8291-9

[27] Hong C.C.; Sevoian M. Interferon production and host resistance to type II avian (Marek's) leukosis virus (JM strain). Appl Microbiol. 1971 11;22(5):818–820. 4332041 10.1128/am.22.5.818-820.1971 PMC376425

[28] Lowenthal J.W.; York J.J.; O'Neil T.E.; Rhodes S.; Prowse S.J.; Strom D.G.; Digby M.R. In vivo effects of chicken interferon-gamma during infection with Eimeria. J Interferon Cytokine Res. 1997 9;17(9):551–558. doi: 10.1089/jir.1997.17.551 9335433 10.1089/jir.1997.17.551

[29] Bharti D, Kumar A, Mahla RS, Kumar S, Ingle H, Shankar H, Joshi B, Raut AA, Kumar H. The role of TLR9 polymorphism in susceptibility to pulmonary tuberculosis. Immunogenetics. 2014 12;66(12):675–81 doi: 10.1007/s00251-014-0806-1 25248338 10.1007/s00251-014-0806-1

[30] Nguyen CT, Kim EH, Luong TT, Pyo S, Rhee DK. ATF3 confers resistance to pneumococcal infection through positive regulation of cytokine production. J Infect Dis. 2014 12 1;210(11):1745–54 doi: 10.1093/infdis/jiu352 24951825 10.1093/infdis/jiu352

[31] Kaspers B.; Lillehoj H.S.; Jenkins M.C.; Pharr G.T. Chicken interferon-mediated induction of major histocompatibility complex class II antigens on peripheral blood monocytes. Vet Immunol Immunopathol. 1994 12;44(1):71–84 7536986 10.1016/0165-2427(94)90170-8

[32] Hu X.; Chen J.; Wang L.; Ivashkiv L.B. Crosstalk among Jak-STAT, Toll-like receptor, and ITAM-dependent pathways in macrophage activation. J Leukoc Biol. 2007 8;82(2):237–243. doi: 10.1189/jlb.1206763 17502339 10.1189/jlb.1206763

[33] Hu X.; Chakravarty S.D.; Ivashkiv L.B. Regulation of interferon and Toll-like receptor signaling during macrophage activation by opposing feedforward and feedback inhibition mechanisms. Immunol Rev. 2008 12;226:41–56. doi: 10.1111/j.1600-065X.2008.00707.x 19161415 10.1111/j.1600-065X.2008.00707.x PMC2630590

[34] Darmani H.; Parton J.; Harwood J.L.; Jackson S.K. Interferon-gamma and polyunsaturated fatty acids increase the binding of lipopolysaccharide to macrophages. Int J Exp Pathol. 1994 10;75(5):363–368. 7999637 PMC2001866

[35] Mita Y.; Dobashi K.; Shimizu Y.; Nakazawa T.; Mori M. Toll-like receptor 2 and 4 surface expressions on human monocytes are modulated by interferon-gamma and macrophage colony-stimulating factor. Immunology letters. 2001 9 3;78(2):97–101. 11672593 10.1016/s0165-2478(01)00241-3

[36] Dalpke A.H.; Eckerle S.; Frey M.; Heeg K. Triggering of Toll-like receptors modulates IFN-gamma signaling: involvement of serine 727 STAT1 phosphorylation and suppressors of cytokine signaling. Eur J Immunol. 2003 7;33(7):1776–1787. doi: 10.1002/eji.200323621 12811837 10.1002/eji.200323621

[37] Schroder K.; Lichtinger M.; Irvine K.M.; Brion K.; Trieu A.; Ross I.L.; Ravasi T.; Stacey K.J.; Rehli M.; Hume D.A.; Sweet M.J. PU.1 and ICSBP control constitutive and IFN-gamma-regulated Tlr9 gene expression in mouse macrophages. J Leukoc Biol. 2007a 6;81(6):1577–1590. 17360957 10.1189/jlb.0107036

[38] Geissmann F, Manz MG, Jung S, Sieweke MH, Merad M, Ley K. Development of monocytes, macrophages, and dendritic cells. Science. 2010 2 5;327(5966):656–61. doi: 10.1126/science.1178331 20133564 10.1126/science.1178331 PMC2887389

[39] Haskó G, Pacher P, Deitch EA, Vizi ES. Shaping of monocyte and macrophage function by adenosine receptors. Pharmacol Ther. 2007 2;113(2):264–75 doi: 10.1016/j.pharmthera.2006.08.003 17056121 10.1016/j.pharmthera.2006.08.003 PMC2228265

[40] Haskó G, Csóka B, Németh ZH, Vizi ES, Pacher P. A(2B) adenosine receptors in immunity and inflammation. Trends Immunol. 2009 6;30(6):263–70. doi: 10.1016/j.it.2009.04.001 19427267 10.1016/j.it.2009.04.001 PMC2756472

[41] Drechsler Y.; Bohls R.L.; Smith R.; Silvy N.; Lillehoj H.; Collisson E.W. An avian, oncogenic retrovirus replicates in vivo in more than 50% of CD4+ and CD8+ T lymphocytes from an endangered grouse. Virology. 2009 4 10;386(2):380–386. doi: 10.1016/j.virol.2009.01.027 19237181 10.1016/j.virol.2009.01.027

[42] Drechsler Y, Tkalcic S, Saggese MD, Shivaprasad HL, Ajithdoss DK, Collisson EW. A DNA vaccine expressing ENV and GAG offers partial protection against reticuloendotheliosis virus in the prairie chicken (Tympanicus cupido). J Zoo Wildl Med. 2013 6;44(2):251–61. doi: 10.1638/2011-0229R1.1 23805542 10.1638/2011-0229R1.1

[43] Crippen T.L.; Sheffield C.L.; He H.; Lowry V.K.; Kogut M.H. Differential nitric oxide production by chicken immune cells. Dev Comp Immunol. 2003 Jun-Jul;27(6–7):603–610. 12697316 10.1016/s0145-305x(03)00035-1

[44] Singh S.; Toro H.; Tang D.C.; Briles W.E.; Yates L.M.; Kopulos R.T.; Collisson E.W. Non-replicating adenovirus vectors expressing avian influenza virus hemagglutinin and nucleocapsid proteins induce chicken specific effector, memory and effector memory CD8(+) T lymphocytes. Virology. 2010b 9 15;405(1):62–69. 20557918 10.1016/j.virol.2010.05.002 PMC2930022

[45] He H, Genovese KJ, Nisbet DJ, Kogut MH. Profile of Toll-like receptor expressions and induction of nitric oxide synthesis by Toll-like receptor agonists in chicken monocytes. Mol Immunol. 2006 3;43(7):783–9. doi: 10.1016/j.molimm.2005.07.002 16098593 10.1016/j.molimm.2005.07.002

[46] Chan G, Bivins-Smith ER, Smith MS, Smith PM, Yurochko AD. Transcriptome analysis reveals human cytomegalovirus reprograms monocyte differentiation toward an M1 macrophage. J Immunol. 2008 7 1;181(1):698–711. 18566437 10.4049/jimmunol.181.1.698 PMC2614917

[47] Gautier EL, Ivanov S, Williams JW, Huang SC, Marcelin G, Fairfax K, Wang PL, Francis JS, Leone P, Wilson DB, Artyomov MN, Pearce EJ, Randolph GJ. Gata6 regulates aspartoacylase expression in resident peritoneal macrophages and controls their survival. J Exp Med. 2014 7 28;211(8):1525–31. doi: 10.1084/jem.20140570 25024137 10.1084/jem.20140570 PMC4113942

[48] Nisole S, Stoye JP, Saib A. TRIM family proteins: retroviral restriction and antiviral defence. *Nat*. *Rev*. *Microbiol*. 2005 3: 799–808. doi: 10.1038/nrmicro1248 16175175 10.1038/nrmicro1248

[49] Si Z, Vandegraaff N, O’Huigin C, Song B, Yuan W, Xu C, Perron M, Li X, Marasco WA, Engelman A, et al Evolution of a cytoplasmic tripartite motif (TRIM) protein in cows that restricts retroviral infection. *Proc*. *Natl*. *Acad*. *Sci*. *USA* 2006 103: 7454–7459. doi: 10.1073/pnas.0600771103 16648259 10.1073/pnas.0600771103 PMC1464360

[50] van Hateren A, Carter R, Bailey A, Kontouli N, Williams AP, Kaufman J, Elliot T. A mechanistic basis for the co-evolution of chicken tapasin and major histocompatibility complex class I (MHC I) proteins. J Biol Chem 2013 288: 32797–32808 doi: 10.1074/jbc.M113.474031 24078633 10.1074/jbc.M113.474031 PMC3820913

